# Deep proteome profiling of the hippocampus in the 5XFAD mouse model reveals biological process alterations and a novel biomarker of Alzheimer’s disease

**DOI:** 10.1038/s12276-019-0326-z

**Published:** 2019-11-15

**Authors:** Dong Kyu Kim, Dohyun Han, Joonho Park, Hyunjung Choi, Jong-Chan Park, Moon-Yong Cha, Jongmin Woo, Min Soo Byun, Dong Young Lee, Youngsoo Kim, Inhee Mook-Jung

**Affiliations:** 1 Department of Biomedical Sciences, Seoul National University, College of Medicine, Seoul, Korea; 2 Proteomics Core Facility, Transdisciplinary Research and Collaboration, Biomedical Research Institute, Seoul National University Hospital, Seoul, Korea; 3 Interdisciplinary Program for Bioengineering, Seoul National University, College of Engineering, Seoul, Korea; 4 Interdisciplinary Graduate Program in Genetic Engineering, Seoul National University, Seoul, Korea; 5 LG Chem Life Science R&D Campus, Drug Discovery Center, Seoul, Korea; 6 Institute of Human Behavioral Medicine, Medical Research Center, Seoul National University, Seoul, Korea; 7 Department of Psychiatry, Seoul National University College of Medicine, Seoul, Republic of Korea; 8 Department of Neuropsychiatry, Seoul National University Hospital, Seoul, Korea

**Keywords:** Alzheimer's disease, Proteomic analysis

## Abstract

Alzheimer’s disease (AD), which is the most common type of dementia, is characterized by the deposition of extracellular amyloid plaques. To understand the pathophysiology of the AD brain, the assessment of global proteomic dynamics is required. Since the hippocampus is a major region affected in the AD brain, we performed hippocampal analysis and identified proteins that are differentially expressed between wild-type and 5XFAD model mice via LC-MS methods. To reveal the relationship between proteomic changes and the progression of amyloid plaque deposition in the hippocampus, we analyzed the hippocampal proteome at two ages (5 and 10 months). We identified 9,313 total proteins and 1411 differentially expressed proteins (DEPs) in 5- and 10-month-old wild-type and 5XFAD mice. We designated a group of proteins showing the same pattern of changes as amyloid beta (Aβ) as the Aβ-responsive proteome. In addition, we examined potential biomarkers by investigating secretory proteins from the Aβ-responsive proteome. Consequently, we identified vitamin K-dependent protein S (PROS1) as a novel microglia-derived biomarker candidate in the hippocampus of 5XFAD mice. Moreover, we confirmed that the PROS1 level in the serum of 5XFAD mice increases as the disease progresses. An increase in PROS1 is also observed in the sera of AD patients and shows a close correlation with AD neuroimaging markers in humans. Therefore, our quantitative proteome data obtained from 5XFAD model mice successfully predicted AD-related biological alterations and suggested a novel protein biomarker for AD.

## Alzheimer’s disease: Protein analysis reveals new biomarker candidate

A protein newly implicated in Alzheimer’s disease could serve as a diagnostic biomarker or therapeutic target. A team led by Youngsoo Kim and Inhee Mook-Jung from Seoul National University, South Korea, analyzed all the proteins expressed in the hippocampus, the brain’s memory center, in mice with and without Alzheimer’s-like disease. They identified more than 1,400 proteins differentially expressed between the mouse model of Alzheimer’s and the normal mice. Among these were 36 secretory proteins that tended to increase their levels along with build-up of amyloid-beta, the protein found in clumps in the brains of people with Alzheimer’s. Many already had known links to Alzheimer’s, but the researchers also identified a novel protein called PROS1. Blood samples from Alzheimer’s patients also showed an increase in PROS1 levels, with a close correlation with amyloid-beta build-up in the brain.

## Introduction

Alzheimer’s disease (AD) accounts for the highest proportion of neurodegenerative disease patients worldwide and is the leading cause of dementia. The distinct pathological hallmarks of AD are extracellular amyloid plaques and intracellular neurofibrillary tangles accompanied by synaptic loss and neuroinflammation in the brain^1^. The hippocampus, which is essential for memory and cognitive function, is vulnerable to these pathological characteristics in AD patients^2^. Amyloid-associated pathology is the first aspect of the disease to appear and increase, preceding the appearance of cognitive dysfunction and memory loss^3^. The amyloid cascade hypothesis suggests that the production of amyloid beta (Aβ) is the initial amyloid-triggered reaction and that it initiates the subsequent development of AD symptoms^4^. Since Aβ causes cellular toxicity in a variety of ways, it is important to understand the molecular pathologic symptoms that occur in response to Aβ. However, the complex molecular pathologies of AD are not yet fully understood. An improved understanding of the dynamic protein expression changes induced by Aβ and amyloid plaques has provided insight into AD pathogenesis and shed light on some pathways that may be altered in AD. Systematic analysis can provide an overview of the pathological phenomena of AD and suggest potential therapeutic targets and novel biomarkers. Here, we examined the hippocampal proteome of 5XFAD mice, which overexpress mutated versions of the human amyloid precursor protein (APP) and human presenilin-1 (PSEN1) and, thus, produce excessive Aβ in their brains. This overproduced Aβ accumulates and forms extracellular amyloid plaques in the hippocampus beginning at an early age in these mice, resulting in the earlier appearance of AD pathogenesis in the hippocampus of these model mice compared to other AD mouse models^5^.

Mass spectrometry (MS)-based quantitative proteomics is a powerful technology that can offer unprecedented insights into complex biological processes and phenotypes^6^. Advances in liquid chromatography coupled to tandem mass spectrometry (LC-MS/MS) have enabled the high-throughput detection and quantification of thousands of proteins in numerous mouse brain tissue samples^7,8^. At present, there are two popular strategies for protein-based quantification: the label-free quantification (LFQ) and isobaric tandem mass tags (iTRAQ, TMT) approaches. In general, quantification approaches based on isobaric chemical tags (e.g., TMT) that target primary amines rely on the measurement of reporter ions detected at the MS2 level after fragmentation and allow multiplexing of up to 10 samples without missing values. We recently employed a TMT approach to quantify ~7000 proteins from mouse hippocampi to define signatures associated with AD pathogenesis^9^. However, TMT strategies have several limitations, including the need for expensive labeling reagents, the risk of incomplete labeling of the proteome, the potential for a low peptide identification rate^10^, and the possibility that ratio compression will be caused by coisolation and co-fragmentation of interfering ions^11^. Although MS3-based approaches have been introduced to circumvent the ratio compression issues^11^, these approaches are usually limited to specialized MS instruments and/or are not yet in routine use^12,13^.

LFQ does not require the incorporation of stable isotopes and instead relies on the comparison of peptide MS1 signal intensities between LC-MS runs; it is therefore easy to integrate into most experimental workflows^14^. LFQ has some limitations, such as an increased risk of missing values and variances for lower-abundance proteins because it measures each sample individually, but advances in detection and quantitation have shown promise in ameliorating the former issue^14,15^. In addition, recent in-depth comparisons of isobaric labeling with LFQ showed that the latter yields superior results in terms of protein coverage and increased protein identification^10,16^.

Several groups have constructed proteome databases from human AD brain samples and AD mouse models^17–19^, but these studies examined fixed time points and, thus, did not reveal progressive proteomic changes induced by the accumulation of Aβ in the brain. In addition, these studies did not address potential age-related changes in the brain proteome, and the observed differences were therefore assumed to be genotype related. Although a few studies have analyzed the hippocampal proteome of AD model mice, they examined the changes in proteins and biological pathways previously known to be associated with AD^8^. Here, we sought to investigate biological pathways altered by changes in age-related protein expression in AD model mice to gain new insight into the pathogenesis of AD. Furthermore, we identified the group of DEPs exhibiting the same increasing tendency as the Aβ production pattern and referred to these proteins as the Aβ-responsive proteome. Based on an in-depth hippocampal proteome database, we discovered a novel biomarker for AD that fulfills two conditions: Aβ-responsiveness and a secretory nature of the protein, reflecting AD pathology in peripheral tissues. Among the proteins belonging to the Aβ-responsive proteome, several secretory proteins involved in the complement and coagulation pathways were identified. Specifically, we identified a new target protein, Vitamin K-dependent protein S (PROS1), which appears to be a novel serum biomarker candidate for AD. Since PROS1 is mainly produced by microglia in the brain, we suggest that PROS1 plays a role in the neuro-inflammatory responses of AD pathogenesis. As the first evidence for assessing the potential pathological relevance of PROS1 in AD, we confirmed the association of PROS1 with AD pathogenesis in mice and humans and showed that it serves as a novel biomarker.

## Materials and methods

### Cell culture

The BV-2, C8-D1A, bEND3, U373, and HT22 cell lines were grown in Dulbecco’s modified Eagle’s medium (DMEM) supplemented with L-glutamine, 10% FBS, and 1% penicillin/streptomycin at 37 °C with 5% CO_2_. The cells were harvested with PBS and flash frozen in liquid nitrogen for storage^20^.

### Culture of primary microglia

The culture of primary microglia was performed as previously described^21^. Primary microglia were cultured from the brains of postnatal day 1–2 ICR mice. Briefly, whole brains without their meninges were dissociated in 10 ml of DMEM containing 10% FBS by using glass pipettes. The resulting dissociated cell suspension was filtered through a 40 μm cell strainer to remove tissue debris. After adding 10 ml of DMEM to the filtered cell suspension, the whole cell suspension was plated in PDL-coated T-75 flasks and incubated at 37 °C with 5% CO. After 2 days, the whole culture medium was replaced with fresh culture media. After 2 weeks, astrocytes were layered at the bottom of the T-75 flask, and microglia grew on top of the astrocyte layer. The flasks were lightly tapped and shaken to detach microglia. The resulting cells were seeded in PDL-coated plates.

### Transgenic mice

5XFAD mice (Tg6799; Jackson Laboratory, Stock #006554) possess five familial AD mutations within two human transgenes, *APP* and *PSEN1*. The human *APP* transgene contains the Swedish (K670N, M671L), Florida (I716V), and London (V717I) mutations, and the human *PSEN1* transgene contains the M146L and L286V mutations. Both genes are regulated by the murine Thy1 promoter. These mice rapidly develop an AD-like pathogenesis, including amyloid plaques, activation of the immune system, and cognitive impairment. The deposition of extracellular amyloid plaques begins at 2 months of age, when it is observed in the fifth layer of the cortex and in the subiculum region. Amyloid plaques are deposited throughout the hippocampus by 4–5 months of age. Neuroinflammation starts at 2 months of age and is followed by the deposition of amyloid plaques. Memory impairment is observed beginning at 6 months of age^5^. To visualize microglia in 5XFAD mice, 5XFAD mice were crossed with CX3CR1^GFP/GFP^ mice (JAX stock #005582, The Jackson Laboratory)^22^. The 5XFAD:CX3CR1^GFP/+^ offspring exhibited AD pathogenesis and expressed GFP in their microglia. The CX3CR1^GFP/+^ offspring, which did not carry the human *App* and *Psen1* mutations, were used as wild-type controls (wild-type:CX3CR1^GFP/+^). All experiments were performed using female mice. All animal experiments and management procedures were performed as outlined in the guidelines of the Institutional Animal Care and Use Committee of Seoul National University.

### Other methods

Additional experimental methods are provided in the Supplementary Materials and Methods.

## Results

### Deep hippocampal proteomic analysis of 5XFAD mice

5XFAD transgenic mice develop AD pathogenesis rapidly in their brains, with amyloid plaques appearing in the hippocampus beginning at 3–4 months of age^5^. To quantify the amyloid plaques deposited in the hippocampus of 5XFAD mice at 5 and 10 months of age, we performed immunohistochemistry using the biotin-4G8 antibody to stain amyloid plaques. At 5 months of age, we observed a few small amyloid plaques in the hippocampus of these model mice. At 10 months of age, the amyloid plaques were increased in both size and number (Supplementary Fig. S1). To investigate the neurodegeneration-associated hippocampal proteome in response to amyloid pathology, we analyzed the hippocampi of 5XFAD mice at 5 and 10 months versus those of wild-type mice.

**Fig. 1:**
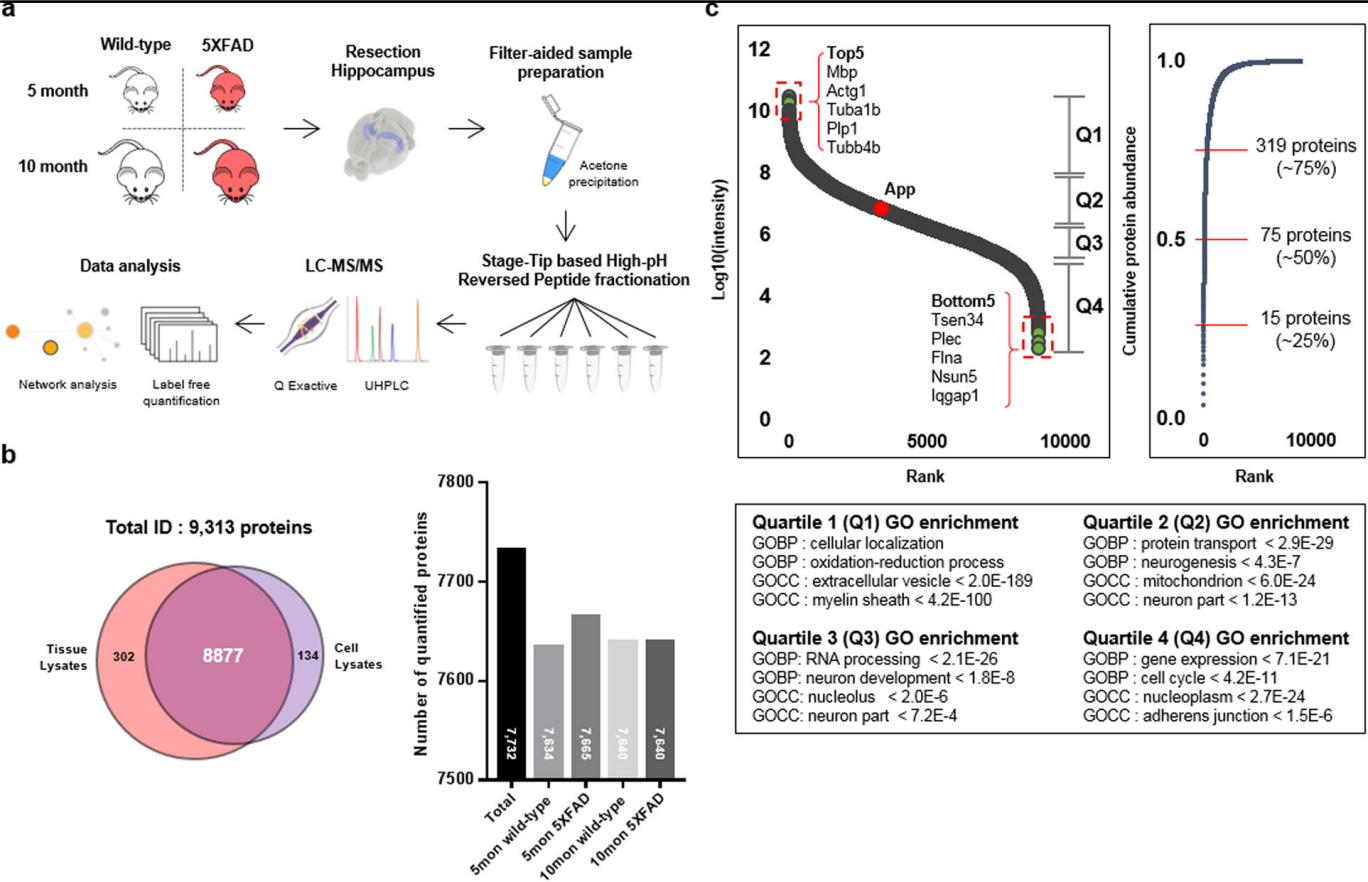
Hippocampal proteome analysis of 5XFAD transgenic model mice. **a** Graphical illustration of the workflow used for our quantitative proteomic analysis. **b** A total of 9313 protein groups were identified in mouse hippocampal samples and mouse cell lines, 7732 of which were quantified in the mouse hippocampal samples. A bar chart showing the numbers of proteins identified in hippocampal samples from each transgenic and wild-type mouse. **c** Dynamic range of protein abundance, spanning seven orders of magnitude. The iBAQ intensities of each sample were used

We performed quantitative proteomic analysis using three replicates of hippocampi dissected from wild-type and 5XFAD mice at 5 and 10 months of age (Fig. 1a). Our group recently showed that double enzyme digestion and peptide-level fractionation coupled to advanced MS instrumentation could achieve protein identification at great depth^9,23^. Building on these findings, we used a combined proteomic strategy including filter-aided sample preparation, high-pH peptide fractionation, and high-resolution Orbitrap mass spectrometry to identify 9313 proteins in the hippocampal proteome (Fig. 1a). To expand the coverage of the identified hippocampal proteome, we used brain-specific cell lines (C8-D1A, BV-2, and HT-22) to generate spectral libraries. In total, we identified 9179 proteins in hippocampal tissues and 9011 proteins in the brain-specific cell lines, among which 8877 proteins (95% of the total) were detected in both systems (Fig. 1b and Supplementary Table S1). We quantified an average of 7600 proteins per mouse group by stringent filtering for valid values (Fig. 1b). We found that the detected protein abundances spanned seven orders of magnitude, with a small subset of 75 proteins constituting 50% of the total protein mass (Fig. 1c). Gene ontology (GO) analysis showed that the GO-Biological Process (GOBP) and GO-Cellular Component (GOCC) terms that were enriched in each quartile presented the expected correlations with the overall protein abundances (Fig. 1c). Interestingly, the high-abundance proteins were strongly enriched for extracellular vesicle proteins. This situation was previously observed in another hippocampus proteome^7^ and presumably indicates that the inter- and intracellular signaling mediated by extracellular vesicles is relevant to cells of the hippocampus. Collectively, our hippocampal proteome data obtained from wild-type and transgenic mice provide a comprehensive resource for the community, including information on the longitudinal changes in almost 9000 proteins.

### Hippocampal proteome remodeling during disease progression in 5XFAD mice

**Fig. 2:**
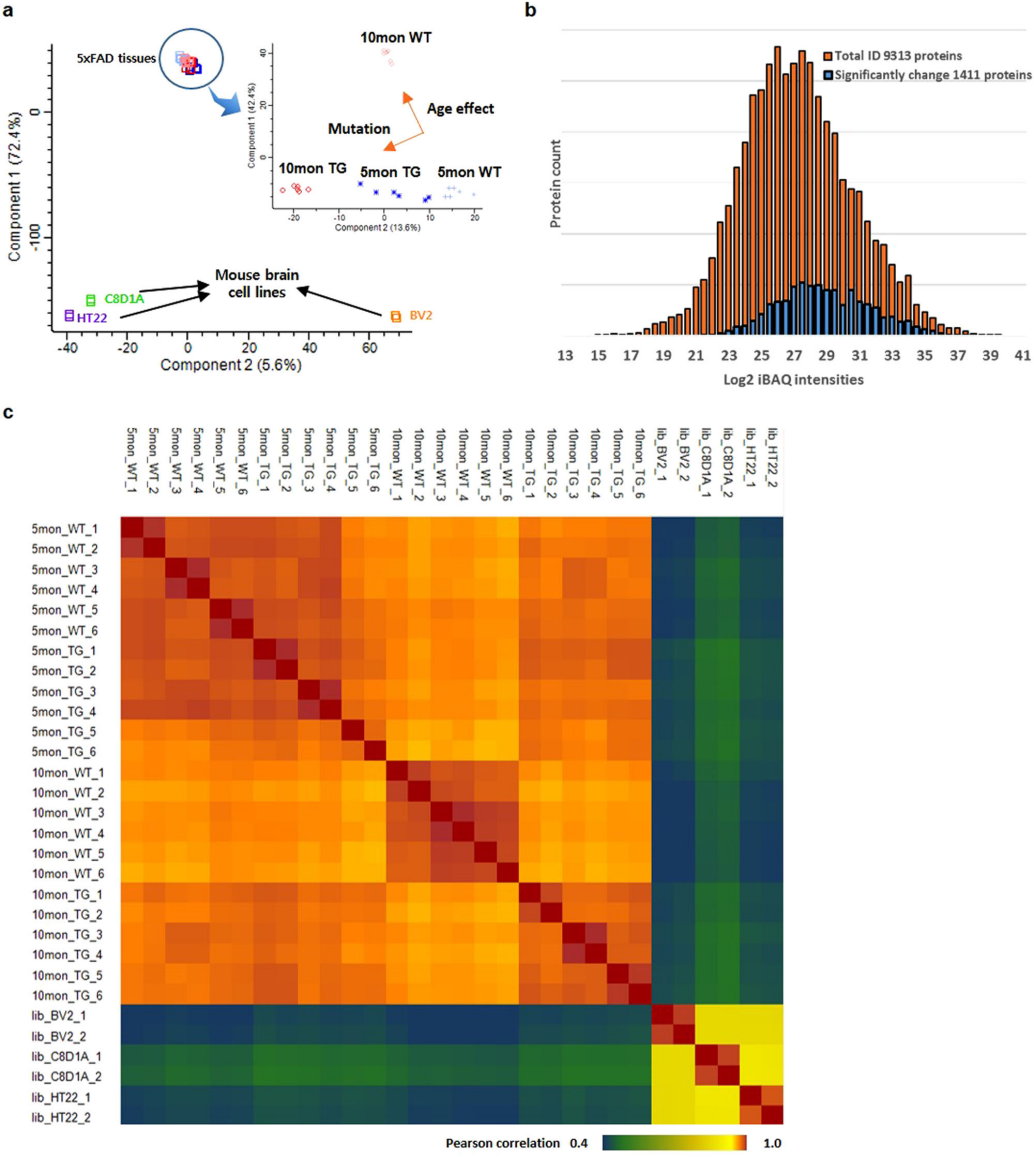
Results of label-free quantification in 5XFAD mice. **a** Principal-component analysis. **b** The distribution of the total identified proteins and differentially expressed proteins (DEPs, blue bar), as indicated by ANOVA with permutations based on an FDR < 0.05. **c** Pearson **c**orrelation of iBAQ intensities across all samples

Principal-component analysis (PCA) of the hippocampal proteomes obtained from 5XFAD and wild-type mice showed clear separation of the samples, with two significant effects being noted (Fig. 2a). First, the samples corresponding to the 5XFAD mouse groups after disease onset (i.e., at 10 months) were separated from the wild-type samples of the same age, indicating that protein expression undergoes extensive remodeling during AD pathogenesis. Second, the proteomes of the 10-month-old mice of both genotypes were separated from those of the corresponding 5-month-old mice (Fig. 2a). Pairwise comparison analysis revealed 531 and 2216 DEPs (*p* < 0.05 and fold change > 1.5) between the wild-type and 5XFAD mice at 5 and 10 months, respectively (Supplementary Fig. S2a, b, and Supplementary Table S2 and S3). The levels of the mutant forms of human APP and PSEN1 were detected as positive controls. These proteins are known to be overexpressed in 5XFAD mice^5^, and the results supported the accuracy of our quantitative analysis. The volcano plots showed an age-dependent increase in DEPs during the progression of amyloid pathology, which is consistent with our previous study^9^.

A hallmark of AD pathology is the aggregation and deposition of misfolded proteins, particularly aggregated Aβ in the form of extracellular senile plaques. The deposition of senile plaques has been linked to several risk genes discovered in genome-wide association studies (GWASs)^24,25^. Here, we tested the connection between putative AD susceptibility genes and the hippocampal proteome of 5XFAD mice. Our screen identified 98 significantly altered proteins that were genetically linked to late-onset AD (LOAD) (Supplementary Fig. S2c and Supplementary Table S4). Various processes and pathways were implicated by the identified proteins. For example, genes involved in the immune response (Abca7, Clu, Mef2c, and Inpp5d) were highly upregulated in 10-month-old 5XFAD mice, indicating that increasing amounts of Aβ in the hippocampus are closely related to the immune response. Genes implicated in lipid metabolism (Apoe, Abca7, and Clu) were upregulated in 10-month-old 5XFAD mice compared with wild-type mice, supporting the idea that a high cholesterol level in midlife can increase an individual’s risk of developing AD late in life^26^. Two other identified proteins, Cd2ap and Picalm, are involved in endocytosis, which is a critical process in synaptic transmission and the response to neural damage. These proteins are also involved in the trafficking of APP between the plasma membrane and endosomes, which plays a key role in AD pathogenesis^27^. Together, these findings show that a large number of proteins genetically associated with AD are altered at the protein level in the hippocampus of this AD-like mouse model.

To identify significant differences between the tested genotypes and time points, we used ANOVA to examine the complete dataset. We extracted 1411 proteins that were significantly different between wild-type and transgenic mice at 5 and 10 months of age (Fig. 2b). Most of these proteins were high- to intermediate-abundance proteins because it is more difficult for low-abundance proteins to reach statistical significance in such an analysis. The high degree of quantitative accuracy offered by our LFQ technique encouraged us to investigate proteomic differences within and between AD-induced hippocampal tissues or immortalized cell lines of mouse brain origin. We examined the correlation between samples to determine whether simple unsupervised analysis would enable us to select DEPs. Overall, the Pearson correlation values between the cell lines and tissues ranged from 0.4 to 0.6, whereas that between hippocampal tissues was 0.97 on average. Unsupervised clustering of the correlations between hippocampal tissue samples showed age-dependent changes in protein abundance, which is consistent with other studies of Alzheimer’s disease animal model (Fig. 2c)^8,28^. We next examined the canonical pathways related to the 1141 DEPs by performing IPA database-anchored enrichment analysis for diseases and functions. The cellular processes found to be significantly activated in 5XFAD mice included processes related to major hallmarks of AD such as neurodegeneration, cell death, and neuronal disorders. The cellular processes that were inactivated in 5XFAD mice included synaptic transmission, long-term potentiation, and neuronal development (Supplementary Fig. S3a–c). Comparative analysis of canonical pathways was performed to identify which canonical pathways were most significantly regulated in the 5XFAD mice of each age and how they changed with disease progression over time (Supplementary Fig. S4). For instance, inactivation of the synaptic long-term potentiation pathway was first observed in transgenic mice at 10 months of age, indicating that this alteration becomes significant in the late stage of the disease. In contrast, epithelial adherens junction remodeling and Fcγ receptor-mediated phagocytosis by macrophages were strongly activated at 5 months of age but not at 10 months of age, indicating that these pathways are early responsive pathways.

### Hierarchical analysis of the 5XFAD hippocampus according to the stages of the disease

**Fig. 3:**
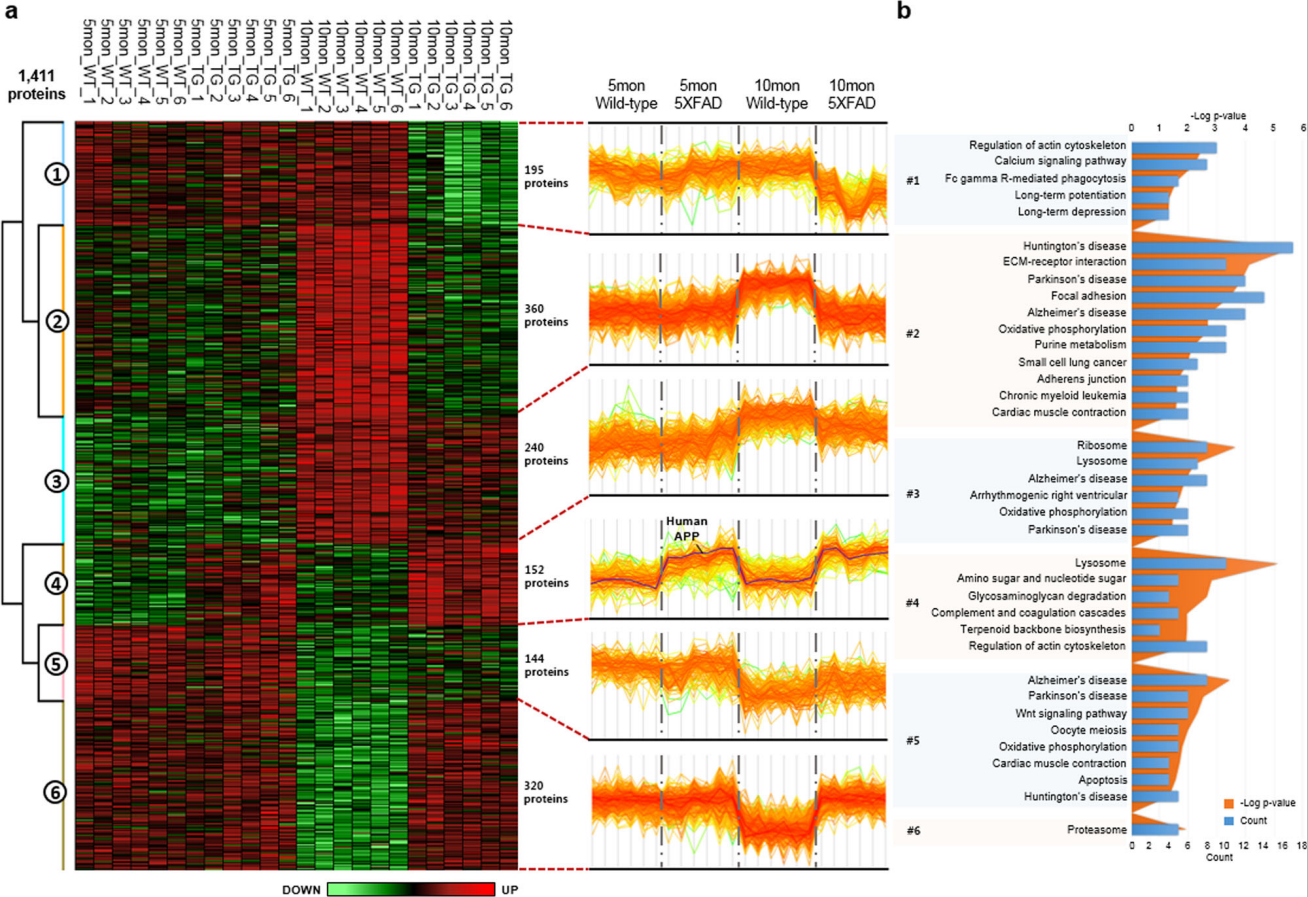
Identification of DEPs related to AD pathogenesis in the hippocampus of 5XFAD mice. **a** Hierarchical clustering of DEPs across the different genotypes and ages (ANOVA, FDR < 0.05). The protein expression level is given as the Z-normalized protein abundance. The DEPs were classified into Clusters 1–6. The color bar represents the gradient of Z-scores normalized protein abundances. **b** Canonical pathway enrichment (*p* < 0.05) for each cluster was performed using Ingenuity Pathway Analysis (IPA)

To identify significant differences between the genotypes and time points, we performed hierarchical clustering analysis. A heatmap of the 1411 DEPs showed a total of six clusters (Clusters 1–6) (Fig. 3a and Supplementary Table S5). The DEPs of Clusters 3 and 5 were altered by the aging of mice. rather than by disease-related changes; those of Cluster 1 were specifically downregulated in 10-month-old 5XFAD mice; and those of cluster 4 were upregulated in 5- and 10-month-old 5XFAD mice compared with their age-matched controls. The last cluster (Cluster 4) contained human APP, which was overexpressed in 5XFAD mice, along with other proteins whose expression levels changed in response to the amyloid pathology. The remaining subgroups, Clusters 2 and 6, showed upregulated patterns in 10-month-old 5XFAD mice compared with wild-type mice, while no difference was observed between the 5-month-old groups. To further explore the cellular processes and pathways represented by Clusters 1–6, we performed enrichment analysis using the IPA database. Fischer’s exact test was used to identify numerous canonical pathways enriched among the DEPs (*p* < 0.05; Fig. 3b). This analysis revealed that several of the DEP clusters were enriched for terms previously associated with AD. Proteins of Clusters 1 and 6, which showed specific up- and downregulation in 10-month-old 5XFAD mice, were associated with processes and components related to calcium signaling (*p* = 1.8E^−3^), long-term potentiation (*p* = 0.8E^−3^), and the proteasome (*p* = 9.8E^−3^). These findings are consistent with those of previous studies^29–33^. Cluster 4, which contained the APP protein, a hallmark of AD pathogenesis, was enriched for processes and components known to be biologically important in AD, including the lysosome (*p* = 2.7E^−6^), glycosaminoglycan degradation (*p* = 1.1E^−3^), and the complement and coagulation cascades (*p* = 6.5E^−3^) (Fig. 3b).

### Network analysis of proteomic changes unique to 5XFAD

**Fig. 4:**
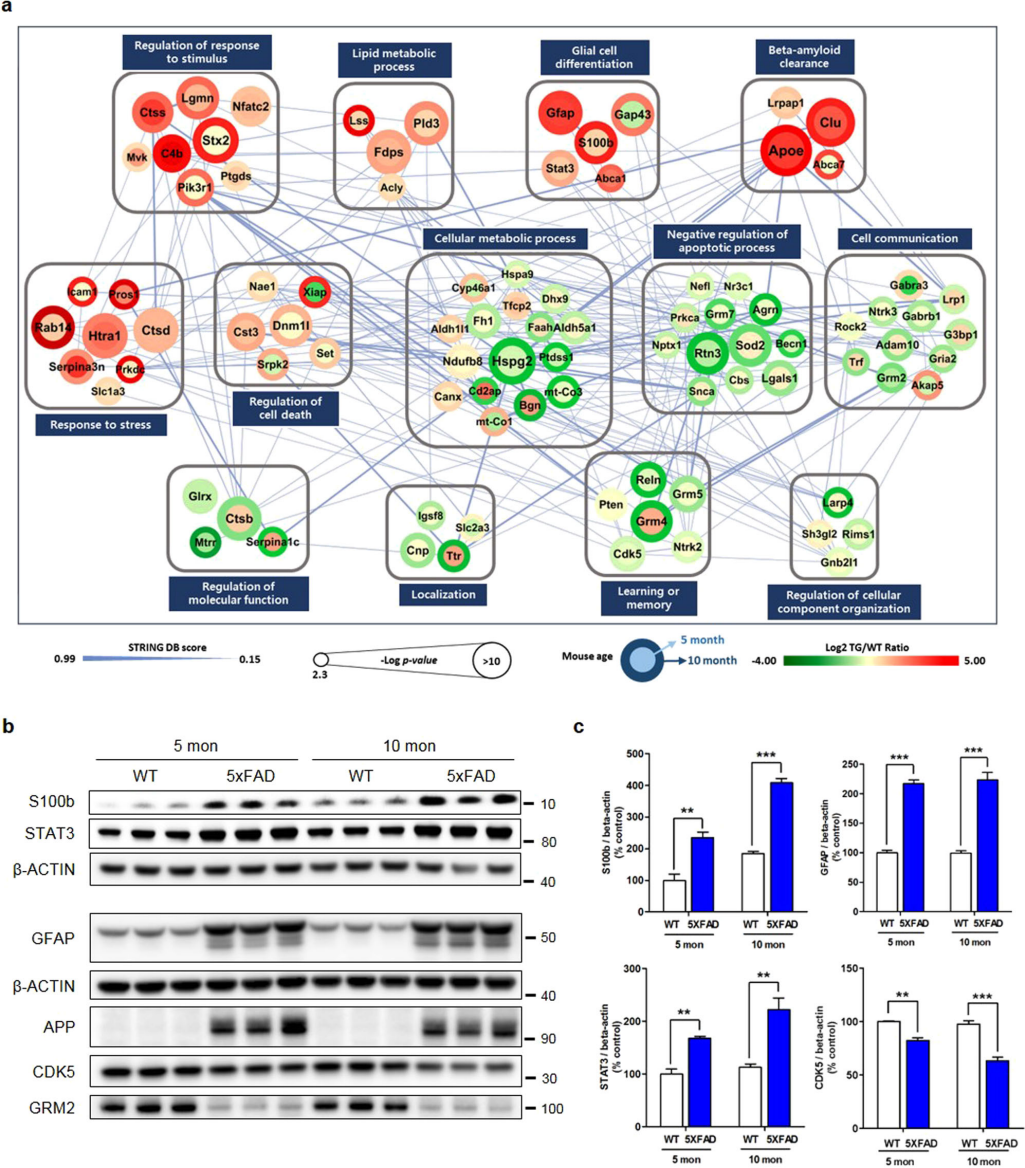
Network analysis of proteins related to AD. **a** The network model describing the protein-protein interactions (PPIs) of 92 AD-related proteins. Node colors represent an increase (red) or decrease (green) in 5-month-old 5XFAD mice (center) and 10-month-old 5XFAD mice (boundary) compared to age-matched wild-type mice. The color bar represents the gradient of log2 protein ratios. The size of a circle represents -log10(P) value, where P is the *p*-value. The edges represent PPIs obtained from the STRING database. **b** The AD-related proteins listed in Fig. 4a were examined by western blotting of hippocampal samples from 5- and 10-month-old wild-type and 5XFAD mice. **c** Quantification of the data shown in Fig. 4b (*n* = 3 per genotype for both ages; two-tailed unpaired *t*-test; **p < 0.01 and ***p < 0.001). The results are expressed as the mean ± SEM

To explore the collective functions of the DEPs in the aforementioned genotype- and age-responsive processes, we reconstructed a network model to describe the interactions among the DEPs. In this network modeling (Fig. 4a), we focused on the 92 AD-associated DEPs that were selected based on the IPA database (Supplementary Fig. S5). The obtained network model showed that there were alterations in pathways associated with the pathogenesis of AD (beta-amyloid clearance, regulation of the response to stimulus, glial cell differentiation, and regulation of apoptosis), suggesting that cellular responses to the neurotoxic effects of Aβ accumulation are activated in the 5XFAD mouse hippocampus. In addition, the network model revealed alterations among various cell communication pathways (cell communication, localization, regulation of cellular component organization, and learning and memory), indicating that Aβ accumulation alters inter- and intracellular communication during AD progression. The collective alterations in the DEPs were represented by 13 GOBPs, suggesting a close association with the biological processes that take place in the brains^19,34^ and plasma^35^ of patients with AD.

To validate the results of our network modeling strategy, we performed western blot analysis in an independent set of mouse hippocampus samples. The overexpression of the human APP protein was verified in the hippocampi of both 10- and 5-month-old 5XFAD mice to confirm the genotype of each mouse. The expression levels of S100b, GFAP, and STAT3 were increased in 5XFAD mice at both 5 and 10 months of age, indicating that continuously produced Aβ induces activated glial inflammation and differentiation. The expression levels of CDK5 and GRM2 were decreased in 5- and 10-month-old 5XFAD mice compared to the controls, providing a mechanistic basis for the synaptic dysfunction and cognitive impairment seen in AD (Fig. 4b, c). To investigate how the 5XFAD mouse model reflects the molecular pathogenesis of human AD, we further performed a comparative analysis of the sets of differentially expressed proteins between healthy aged human brains and the brains of AD patients, which were assembled using a wide range of previous large-scale proteomic studies^19,36–39^. Among almost 1000 proteins showing altered expression associated with pathological and functional decline, 477 were identified in our age-specific expression group (Supplementary Fig. S6). In particular, 71 commonly observed proteins among the human proteome data and the 5-month-old, and 10-month-old DEP groups, such as GFAP, CLU, and APP, were deduced to exhibit significant connections with the pathology of AD (Supplementary Table S6)^40^. In addition, the other proteins in the categories related to neurodegeneration may play a significant role in future studies of AD. Together, the findings generated from our network model suggest that the alterations of the hippocampal proteome observed in 5XFAD mice resemble those observed in human AD patients.

### The Aβ-responsive secretory protein network in the hippocampus

**Fig. 5:**
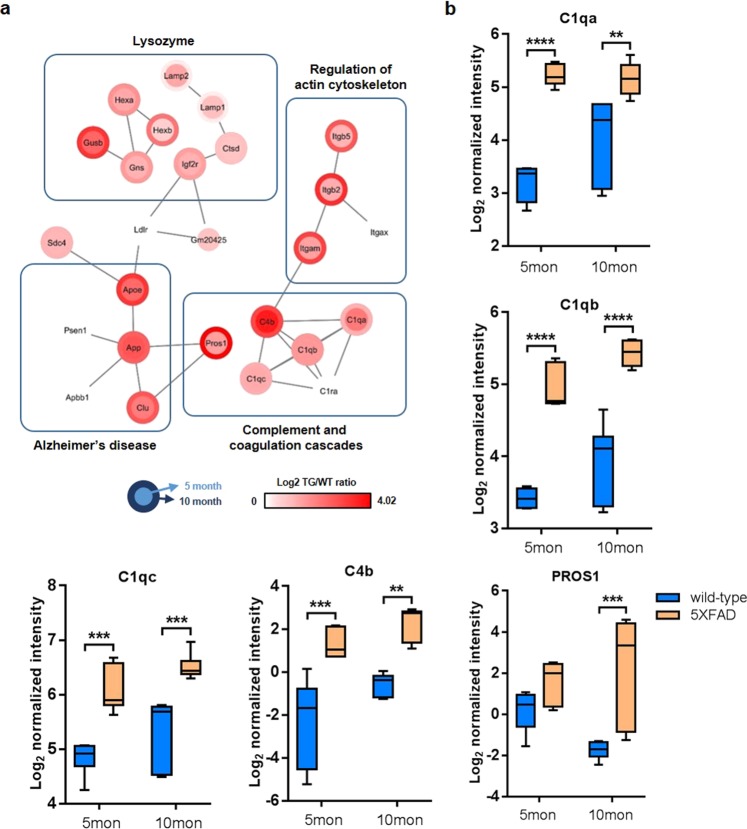
Network analysis of secretory proteins showing that the complement and coagulation cascades are involved in AD. **a** A network model built using the secretory proteins found in Cluster 4 of Fig. 3a. Many of the proteins were grouped into four biological pathways and components: lysozyme, regulation of the actin cytoskeleton, Alzheimer’s disease, and complement and coagulation cascades. **b** Five proteins (PROS1, C4b, C1qa, C1qb, and C1qc) belonging to the complement and coagulation cascades were assessed in 5- and 10-month-old wild-type and 5XFAD mice. The normalized intensity of each protein is presented in the boxplots. The central rectangle represents the first and third quartiles; the central line inside the box represents the mean; and the whiskers extend to the minimum and maximum values. Comparisons were performed via two-way ANOVA. **p < 0.01, ***p < 0.001, and ****p < 0.0001

Using the hierarchical cluster map of the hippocampal proteome of 5XFAD mice organized by aging and the disease, we set out to identify the proteins that had not been extensively investigated in relation to AD but showed potential to be useful serum biomarkers for AD. We performed a bioinformatic analysis in which we selected 1) proteins that tended to increase with the accumulation of Aβ (i.e., those of Cluster 4) and 2) proteins that were predicted to be secreted extracellularly and, thus, could act as biomarkers in serum. Regarding the latter point, various proteins differ in their levels of secretion into the cerebrospinal fluid (CSF) or bloodstream as a disease progresses and, thus, can reflect the progression of the disease^41^. Among the 152 proteins in Cluster 4, SignalP 4.0 software identified 36 as potential secretory proteins (Supplementary Table S7). The network analysis based on protein-protein interactions (PPIs) showed that the selected secretory proteins included several proteins that are involved in lysozyme pathways, the complement and coagulation cascades, and the regulation of the actin cytoskeleton and several well-known AD-associated proteins (APOE, APP, and CLU) (Fig. 5a). Previous reports have suggested that complement proteins are associated with AD pathogenesis^42,43^. Complement proteins, such as C1q and C3, are produced and secreted from microglia to mark targets for phagocytosis^44^. When these complement proteins localize to the synaptic region, microglia expressing complement receptors engulf synapses through the complement pathway. In early AD brains, the secreted complement protein C1q binds to Aβ at a synapse by interacting with various synaptic proteins. Phagocytic microglia easily eliminate these synaptic elements, leading to early synaptic loss in AD^45^. In addition to including several well-known complement proteins, Cluster 4 contained the novel protein PROS1, which is involved in the blood coagulation pathway but has not been investigated in the hippocampus or in association with AD pathogenesis. Here, we found that, consistent with the other complement proteins, PROS1 was increased in the hippocampus of 5XFAD mice at 10 months of age and, thus, is likely to be closely associated with amyloid pathology (Fig. 5b).

### PROS1 is a novel Aβ-responsive protein in AD

**Fig. 6:**
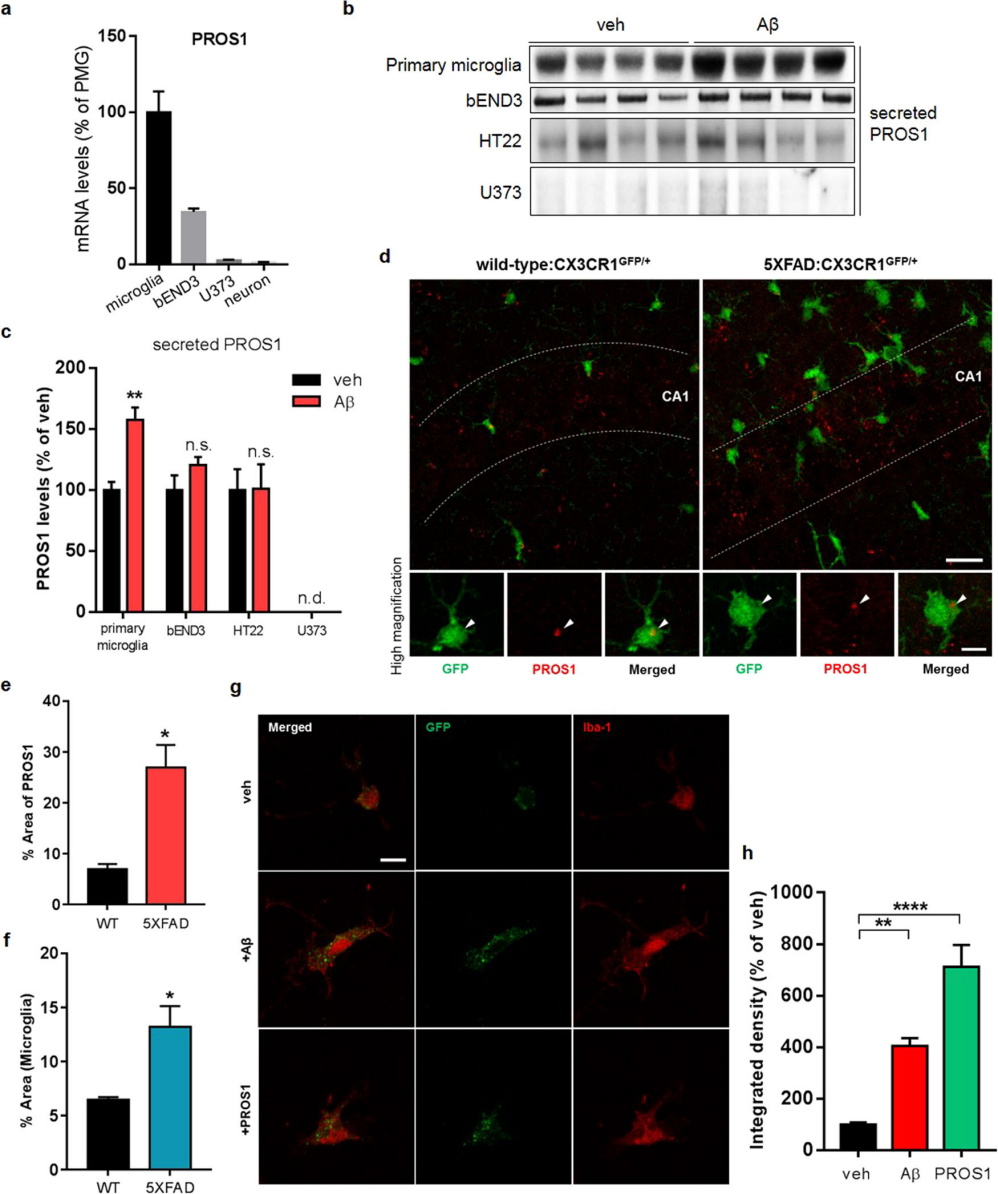
The characteristics of PROS1 in AD. **a** The mRNA levels of PROS1 in different cell types. **b** TCA precipitation followed by western blot analysis confirmed that PROS1 is secreted from primary microglia in response to Aβ (2 μM, 24 h). **c** Quantification of secreted PROS1 (*n* = 4 biologically independent samples; Student’s *t*-test). **d** Immunohistochemical analysis of PROS1 in 10-month-old wild-type: or 5XFAD:CX3CR1^+/GFP^ mice. Arrowheads indicate anti-PROS1 immunopositivity (red) within microglia. Scale bars represent 20 μm and 5 μm (in the high-magnification images). **e** Quantification of the percentage of the area containing PROS1 in the hippocampus (*n* = 3; two-tailed unpaired *t*-test). **f** Quantification of the percentage of area covered by microglia in the hippocampus (*n* = 3; two-tailed unpaired *t*-test). **g** Immunocytochemistry of microglial phagocytosis accelerated by either Aβ (2 μM, 24 h) or PROS1 (30 nM, 24 h) in vitro. The scale bar represents 10 μm. **h** Quantification of the amount of apoptotic cell debris engulfed by primary microglia (*n* = 4; one-way ANOVA). *p < 0.05, **p < 0.01, ****p < 0.0001. The results are expressed as the mean ± SEM

No previous reports have proposed that a quantitative change in the level of PROS1 could be a pathological phenotype in the AD brain. Moreover, the biological functions of PROS1 in the hippocampus and its possible role(s) in AD pathogenesis are unknown. In the mouse hippocampal proteome, since PROS1 appears to be present at a low abundance, it has been difficult to detect PROS1 in other hippocampal proteome databases (Supplementary Fig. S7). *Pros1* was recently identified as a microglia-specific gene in the CNS^46^. It possesses a signal peptide for conventional secretion, suggesting that its cleaved form can be secreted to function in the extracellular environment^47^. To investigate the biological characteristics of PROS1, we first examined its mRNA levels in different brain-relevant cell types. We found differences in *Pros1* mRNA transcript abundance in primary microglia and bEND3 cells (an endothelial cell line that exhibits a much lower expression level compared to primary microglia), while it was present but barely detectable in primary hippocampal neurons and U373 cells (an astrocyte cell line) (Fig. 6a). To further confirm the alteration of PROS1 secretion in response to Aβ, different cell types were treated with Aβ monomers (2 μM) for 24 h, and the amount of secreted PROS1 in cell-conditioned media that had been precipitated by trichloroacetic acid (TCA) to concentrate the proteins present in the cell media was examined. Consistent with the *Pros1* mRNA data, PROS1 was secreted at the highest level from primary microglia (Fig. 6b), and Aβ treatment increased the secretion of PROS1 from primary microglia but not the other tested cell types (Fig. 6c). To investigate whether the transcription of *Pros1* was regulated by Aβ, the mRNA levels of *Pros1* in primary microglia and bEND3 cells treated with Aβ were examined by real-time PCR. The transcription of *Pros1* was not affected by Aβ in either cell type, indicating that Aβ induces the secretion of PROS1, not the production of PROS1, in microglia (Supplementary Fig. S8).

To move beyond our in vitro observations, we used immunohistochemistry to investigate the distribution of PROS1 in the mouse hippocampus. We visualized microglia by using 10-month-old wild-type or 5XFAD:CX3CR1^GFP/+^ mice in which microglia and monocytes in the brain specifically express GFP under the control of the *Cx3cr1* locus. We observed PROS1 inside microglia and in the extracellular environment of the hippocampus (Fig. 6d). Consistent with the ability of Aβ to increase the secretion of PROS1 from microglia in vitro, the amount of PROS1 localized outside of microglia was increased in the 5XFAD:CX3CR1^GFP/+^ mouse hippocampus compared to that of the corresponding wild-type:CX3CR1^GFP/+^ mice (Fig. 6e). Since Aβ causes microgliosis as one of the pathological characteristics of AD^48^, the area occupied by microglia was increased in the 5XFAD:CX3CR1^GFP/+^ mouse hippocampus (Fig. 6f), indicating that the secretion of PROS1 is closely related to microglial activation.

In the blood serum, PROS1 has been shown to function as an opsonin to facilitate the phagocytosis of macrophages following binding to phosphatidylserine on apoptotic cell debris^49^. To verify the biological functions of PROS1 in AD pathogenesis, we investigated the phagocytic ability of microglia in the presence of Aβ or PROS1 in vitro. The role of PROS1 in stimulating microglial phagocytosis was examined by treating apoptotic cell debris expressing GFP in primary microglia with Aβ (2 μM) or PROS1 (30 nM) for 24 h. We found that GFP-positive phagosomes were localized within the microglial cytoplasm (Fig. 6g), and treatment with Aβ or PROS1 greatly increased the phagocytosis of microglia (Fig. 6h). Taken together, the results indicated that PROS1 secretion from microglia induced by Aβ facilitates phagocytic activity to eliminate apoptotic cells in the pathogenesis of AD.

### Serum PROS1 reflects the progression of AD pathologies in 5XFAD mice and human AD patients

**Fig. 7:**
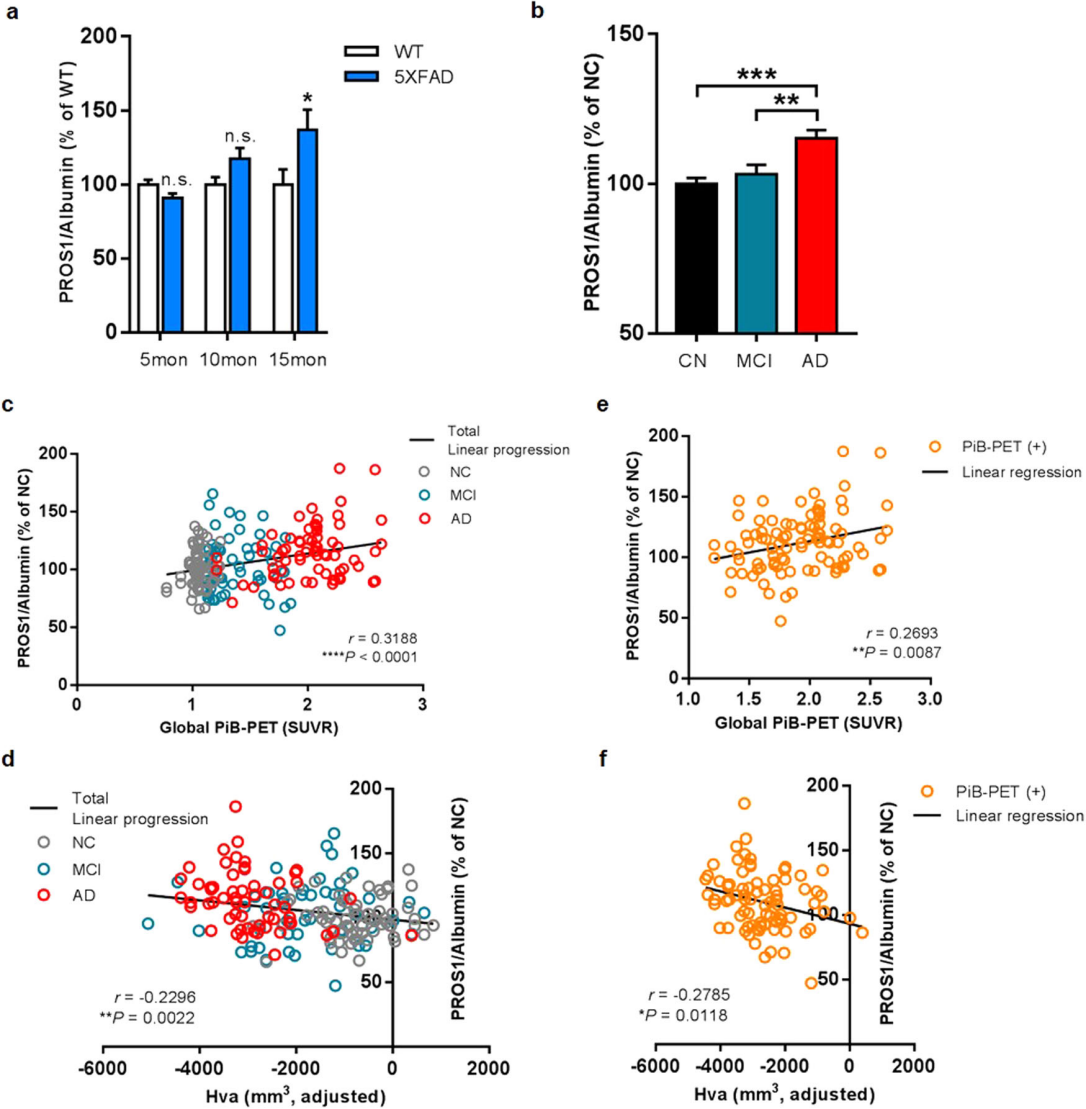
Serum PROS1 correlates with AD pathologies in 5XFAD mice and human AD patients. **a** Serum PROS1 levels in 5-, 10-, and 15-month-old wild-type and 5XFAD mice were measured by western blot analysis (*n* = 7 per genotype for 5- and 10-month-old and *n* = 7/6 for 15-month-old wild-type and 5XFAD mice; Student’s *t*-test). **b** Differences in serum PROS1 levels between cognitively normal (CN) subjects and those with mild cognitive impairment (MCI) and AD dementia (*n* = 67 for NC, *n* = 60 for MCI, and *n* = 68 for AD; one-way ANOVA). **c, d** Serum PROS1 levels correlate with global PiB-PET (global amyloid retention) and the adjusted hippocampal volume area (Hva) in the total participants. **e, f** Serum PROS1 levels correlate with global PiB-PET and hippocampal volume in PiB-PET (+) subjects (*n* = 94 for global PiB-PET and *n* = 81 for Hva). *p < 0.05, **p < 0.01, ***p < 0.001, and ****p < 0.0001. The results are expressed as the mean ± SEM

The abundance or activity of other complement proteins (e.g., C3 and C5) measured directly in CSF or blood serum samples has been reported to reflect disease progression as a biomarker of AD^50–52^. Thus, we hypothesized that PROS1 in sera derived from hippocampi can reflect AD pathogenesis and serves as a potential serum biomarker of AD. To evaluate the effectiveness of serum PROS1 levels as a serum biomarker for AD pathogenesis in the brain, we examined serum PROS1 levels in 5XFAD mice. The levels of serum PROS1 in 5XFAD mice were significantly increased with age compared with those in wild-type mice, indicating that severe AD pathogenesis in the brain can be reflected by serum PROS1 levels (Fig. 7a and Supplementary Fig. S9a). To investigate the tissue origin of increased serum PROS1 levels, we first examined PROS1 levels in the liver, in which most of the serum PROS1 present is synthesized by hepatocytes^53^. There was no significant difference between the 10- and 15-month-old wild-type and 5XFAD mice, suggesting that the production of serum PROS1 by the liver did not affect the increase observed in 5XFAD mice (Supplementary Fig. S9b, c). We depleted hippocampal microglia by injecting clodronate liposomes into 15-month-old mouse hippocampi to investigate whether the alteration of serum PROS1 levels derived from hippocampal microglia. The administration of clodronate liposomes induced a decrease in the number of hippocampal microglia beginning 2 days after intrahippocampal injection (Supplementary Fig. S10a). At 6 days after the injection of clodronate liposomes, serum PROS1 levels were significantly decreased in 5XFAD mice compared to 5XFAD mice injected with control liposomes (Supplementary Fig. S10b), indicating that the secretion of PROS1 from microglia affects serum PROS1 levels, reflecting the pathogenesis of AD.

Given that serum PROS1 levels reflect AD pathogenesis in 5XFAD mice, we compared the abundance of PROS1 in serum between cognitively normal (CN), mild cognitive impairment (MCI), and AD dementia groups via western blot analysis. The characteristics of the participants are described in Table 1 (*n* = 195; 67 CN, 60 MCI, 68 AD). Indeed, the levels of PROS1 in the AD dementia group were significantly higher than those in both the CN and MCI groups (Fig. 7b). Furthermore, we conducted correlation analyses between serum PROS1 levels and two AD neuroimaging biomarkers: global amyloid retention according to [^11^C] Pittsburgh compound B (PiB)-positron emission tomography (PET) and the adjusted hippocampal volume (HVa) according to magnetic resonance imaging. Among all subjects, serum PROS1 levels showed significant correlations with both global amyloid retention (global PiB-PET) and HVa (Fig. 7c and d). In addition, these correlations were consistent in PiB (+) subjects who possessed amyloid pathology in their brain (Fig. 7e, f). The results suggest that the increase in serum PROS1 levels is a potential blood biomarker for predicting in vivo AD pathologies in the brain.

**Table 1:** Demographic characteristics of participants

	Total, *n* = 195			
Demographics (*n*)	CN (67)	MCI (60)	AD (68)	*P*-value
Gender, M/F, *n*	35/32	20/40	22/46	
Age, years, mean ± SEM	68.6 ± 1.0	73.88 ± 0.9	72.72 ± 0.9	<0.001^a^
Education, mean ± SEM	11.43 ± 0.6	9.78 ± 0.6	10.25 ± 0.7	0.16^a^
MMSE raw score, mean ± SEM	26.72 ± 0.3	22.8 ± 0.4	16.57 ± 0.5	<0.0001^a^
MMSE z-score, mean ± SEM	0.22 ± 0.1	−1.03 ± 0.1	−3.31 ± 0.2	<0.0001^a^
ApoE4 positivity, ε4+/ε4−	10/57	17/43	40/28	<0.0001^b^

Data were presented as mean ± SEM or *n*

*CN*  cognitively normal, *MCI* mild cognitive impairment, *AD* Alzheimer’s disease, MMSE z-score, mini-mental state examination with the correction for age, sex, and education level; ApoE, Apolipoprotein ε4

^a^*P*, *p*-values from ANOVA with Tukey’s post-hoc test

^b^*P*, *p*-values from chi-square test

## Discussion

With increasing advancements in proteomic technologies, the complex brain proteome can now be resolved using mass spectrometry. Importantly, the quantitative analysis of proteomic changes during disease progression has the potential to reveal new candidate biomarkers and/or therapeutic targets for neurodegenerative diseases^54^. Recent studies of progressive neurodegenerative diseases have focused on identifying alterations in the proteome of disease-relevant subregions^54^. These studies have highlighted appropriate quantification strategies that allow researchers to analyze thousands of proteins and answer questions with a spatiotemporal dimension^38,55^. Many of the disease-associated proteins identified in cells of the CNS are mainly involved in membrane-associated processes such as neurotransmitter-based signaling and cell-to-cell communication and are often present at low concentrations. Thus, in-depth proteomic analysis is needed to sensitively investigate small quantitative changes in protein expression. With recent advances in quantitative proteomic technologies, we now have a solid platform for applying in-depth proteomic research to neuroscience^54^. Label-free quantification (LFQ) has benefited significantly from these advances, which have increased the number of proteins that may be quantified for a given time point and/or a limited amount of input material^36,55,56^. In this study, we employed in-depth proteome profiling with LFQ to characterize the hippocampal proteomes of 5XFAD and wild-type mice at a depth of 9300 proteins. Temporal characterization of the hippocampal proteome during AD progression revealed extensive alterations in brain regions vulnerable to AD. Compared with a recently published proteomic resource for the mouse brain^55^, we identified approximately 1500 additional genes (Supplementary Fig. S11a). Our work therefore significantly expands our understanding of the mouse hippocampal proteome. We also compared the efficiency of protein identification with that reported in other hippocampal proteomic studies of transgenic AD mouse models, and the results suggested that our dataset presents an extremely impressive depth of proteome coverage (Supplementary Fig. S11b)^8,9,57–60^. To verify whether the 5XFAD mouse model and age-matched hippocampal proteome data could be applied to further AD studies, we assessed the commonality between the alterations of the proteome in the 5XFAD mouse model and other AD transgenic mouse models, including hAPP transgenic mice with the Swedish and Indiana mutations in human APP and hAPP/PS1 transgenic mice with the Swedish mutation in human APP and human PSEN1 deltaE9^8^. Interestingly, the comparative analysis indicated that our DEPs were largely different from those of other AD mouse models despite comparison under conditions involving the same region and similar time points (Supplementary Fig. S11c). Although proteins that are known to be strongly associated with AD phenotypes such as App, Apoe, and Gfap were commonly altered proteins in the hippocampi in all AD transgenic models, most of our DEPs did not overlap with those identified in other AD transgenic mouse models (Supplementary Fig. S11c). This difference between the models is consistent with other proteomic studies using AD transgenic mouse models^8,9^, which may be caused by differences in the promoters used for transgene expression, the degree of AD pathology or genetic background, or the methods applied^61^.

To comprehensively understand the complex pathogenesis of AD, we need a systematic approach for investigating proteomic changes in the brain. Here, we sought to investigate the molecular mechanisms of AD pathogenesis by characterizing the abnormal alterations of the brain proteome in the selected AD model mouse. First, we analyzed the molecular characteristics of the hippocampal proteome of wild-type and AD model mice. The proteome of 5-month-old 5XFAD mice, which were affected by early amyloid pathology, did not differ significantly from that of the age-matched normal group. However, at 10 months of age, which represents the late stage of the disease, the hippocampal proteome of 5XFAD mice differed considerably from those of the age-matched normal group and the 5-month-old group. This finding indicates that severe amyloid pathology can drive proteomic changes or vice versa. The hippocampal proteomes of 5XFAD mice affected by progressive amyloid pathology were organized by hierarchical clustering, and the biological pathways belonging to each cluster were examined in an effort to explore the association between proteomic changes and AD pathogenesis. Cluster 1, which contained proteins that were downregulated under severe amyloid pathology (in 10-month-old 5XFAD mice), was enriched for proteins related to the regulation of the actin cytoskeleton, calcium signaling, Fc gamma R-mediated phagocytosis, long-term potentiation, and long-term depression. These findings agree with previous reports that Aβ disrupts calcium homeostasis in neurons and results in functional disruption of their networks^32,62,63^.

Cluster 4, which contained proteins whose expression levels increased with disease progression but were not altered by age in wild-type mice, was considered to represent the Aβ-responsive proteome. The upregulation of proteins in the Aβ-responsive proteome correlated with the increase in Aβ in the hippocampus. Therefore, it may reflect some of the underlying molecular mechanisms of AD pathogenesis and suggest potential biomarkers that could be used to monitor disease progression. This cluster included proteins involved in the lysosomal pathway, the complement system, and the coagulation cascade. Since Aβ is continuously produced and accumulated in the AD brain, the proteolytic systems of neurons and glial cells are activated to remove intra- and extracellular protein aggregates. However, Aβ impairs the proteolytic system, and the autolysosomal system is activated as a compensatory mechanism^64,65^. The complement proteins modulate microglial phagocytosis for synaptic pruning and promote the clearance of apoptotic cells from the brain^66,67^. The process of complement-mediated microglial phagocytosis appears to be closely related to AD pathogenesis^68^. The complement factors C1q and C3 are produced in and released from microglia; they attach to Aβ bound to synapses and target those synapses for elimination, resulting in early synaptic loss in AD mouse models^45^. Our results therefore identify a series of processes ranging from phagocytosis to proteolytic degradation, which may be involved in the pathogenesis of AD.

In an effort to identify a potential new serum biomarker that could be used to identify and/or monitor AD pathogenesis in the brain, we investigated the secretory proteins belonging to the Aβ-responsive proteome. This group included proteins involved in the lysozyme, complement, and coagulation pathways. In addition to complement proteins, we identified PROS1 as a novel target protein that is also involved in the coagulation pathway whose expression levels increase with disease progression. This is the first report that PROS1 is associated with the pathology of AD. PROS1 is fairly well established as an anticoagulant plasma protein that is required for the function of activated protein C (aPC), which results in degradation of the coagulant factors FVa and FV IIIa^69,70^. Unlike the complement proteins, another property of PROS1 is its attachment to negatively charged phospholipids. During apoptosis, cells can no longer control the distribution of phospholipids, causing negatively charged phospholipids (e.g., phosphatidylserine) to be redistributed to the outer surface of the cell membrane^71,72^. PROS1 attaches to the outer membrane of an apoptotic cell and signals for phagocytes to remove the dying cell^73^. PROS1 is recognized by the TAM family receptors (e.g., Tyro3, Axl, and Mertk) on the plasma membrane of phagocytes engulfing apoptotic cells^74^. Recently, it has been reported that PROS1 and the TAM family receptors are highly expressed in adult microglia, suggesting that PROS1 released from microglia can trigger both microglia and astrocytes expressing TAM family receptors to perform phagocytosis via the TAM systems^46,75,76^. Thus, we considered it possible that increases in complement proteins and PROS1 in 5XFAD mice represent distinct pathways of AD pathogenesis. Since the increase in PROS1 identified in our data indicated that apoptosis-associated pathways are upregulated in the hippocampus of 5XFAD mice and that the neuronal apoptotic pathway is more suitable for our hypothesis, PROS1 was the focus of further study.

Here, we report that Aβ induces primary microglia to secrete PROS1 in vitro. However, the same effect was not observed in the mouse endothelial cell line bEND3, which also expresses PROS1. In the mouse hippocampus in vivo, the amount of PROS1 secreted outside of microglia was increased in the hippocampus of 5XFAD mice. In addition, we investigated the possible role of PROS1 in AD pathogenesis, which stimulates microglia to engulf apoptotic cell debris in vitro. Our results suggest that Aβ induces excessive activation of microglia, which triggers the release of PROS1 and the subsequent phagocytosis that characterizes the pathogenesis of AD.

Proteins belonging to the complement cascade have been implicated not only in AD pathogenesis but also as potential blood biomarkers for AD^52,77,78^. Accordingly, we examined whether serum PROS1 levels differed between the cognitively normal, MCI, and AD dementia groups. Consistent with the results obtained in 5XFAD model mice, the levels of serum PROS1 were significantly increased in the AD dementia group. Moreover, two neuroimaging biomarkers, global amyloid retention and hippocampal volume area, were highly correlated with serum PROS1 levels in both the AD dementia group and PiB-PET(+) subjects, indicating that both amyloid pathology and hippocampal atrophy due to neuronal death could be represented by serum PROS1 levels. Since PROS1 is expressed at a relatively low abundance in the hippocampus, in-depth proteomic analysis was required to reveal its existence in this tissue and its alteration under AD. Going forward, it will be useful to identify additional low-abundance brain proteins that exhibit important biological functions and have implications for disease research.

In summary, we revealed biological pathways that are altered in the hippocampus of the 5XFAD mouse model during disease progression. The changes in the hippocampal proteome are relevant to the alterations observed in the human AD brain, indicating that our data recapitulate the pathology of human AD. We identified the Aβ-responsive proteome comprising proteins that are progressively regulated by Aβ. Increases in proteins of the complement system and lysosome pathway coincide with continuous production of Aβ in the hippocampus, indicating that Aβ causes microglial activation and aberrant complement-driven phagocytosis, with subsequent upregulation of the degradation system as the disease progresses. Within the Aβ-responsive proteome, we examined secretory proteins as potential blood biomarkers for predicting AD pathogenesis in the brain. We showed that the PROS1 protein, which is involved in the complement and coagulation pathways, is increased in the hippocampi and sera of 5XFAD model mice and humans with AD. In addition, since serum PROS1 levels are correlated with AD neuroimaging markers, PROS1 serves as a novel serum biomarker for AD. Together, our findings emphasize that in-depth proteomic analysis is useful for pathological analysis and the elucidation of novel target proteins expressed at low abundance in a specific brain tissue.

## Supplementary information

Supplementary materials and methods

Supplementary figure

Supplementary Table S1

Supplementary Table S2

Supplementary Table S3

Supplementary Table S4

Supplementary Table S5

Supplementary Table S6

Supplementary Table S7
