# Supplementary material for: Deep proteome profiling of the hippocampus in the 5XFAD mouse model reveals biological process alterations and a novel biomarker of Alzheimer’s disease
Source: Exp Mol Med. 2019 Nov 15;51(11):136. doi: 10.1038/s12276-019-0326-z (PMC6856180; doi:10.1038/s12276-019-0326-z)
Supplement: Supplementary file 1 — Supplementary materials and methods [file 12276_2019_326_MOESM1_ESM.docx]

# Supplementary Materials and Methods

**Reagents and materials**

The reducing agent-compatible bicinchoninic acid (BCA) assay kit, Tris (2-carboxyethyl) phosphine (TCEP), and LC/MS-grade solvents, including acetone, acetonitrile (ACN), and water, were purchased from Thermo Fisher Scientific (Waltham, MA). Other reagents and materials were purchased from the following companies: dithiothreitol (DTT) and urea from AMRESCO (Solon, OH), sodium dodecyl sulfate (SDS) and Trizma base from USB (Cleveland, OH), sequencing-grade modified trypsin from Promega Corporation (Madison, WI), POROS20 R2 beads from Applied Biosystems (Foster City, CA), C18 Empore disk membranes from 3M (Bracknell, UK). Unless otherwise indicated, all other reagents were purchased from Sigma-Aldrich (St. Louis, MO).

**Sample preparation for MS analysis**

Cells and tissues were lysed at room temperature in lysis buffer (4% SDS, 1 mM TCEP, 0.1 M Tris-Cl, pH 7.4) with 1 min of proved-tip sonication. The lysates were boiled in a water bath at 100°C, centrifuged at 15,000 rpm for 10 min, and passed through a 0.22-μm filter (Spin-X filter; Costar, NY, USA) to remove remnants of the lysed tissue. The concentration of proteins in each supernatant was determined by the absorbance at 562 nm and estimated with respect to a standard curve generated using bovine serum albumin. Tissue lysates (300 ug of protein) were subjected to acetone precipitation. Briefly, five-fold volume of cold acetone were mixed with the lysate, and the mixture was incubated at -20°C for 4 hrs. The precipitated protein pellet was washed with fresh acetone and dissolved in 50 ul of reduction buffer (4% SDS, 0.1 M DTT, 0.1 M Tris-Cl, pH 7.4). The cell lysate (which was not subjected to acetone precipitation) was also mixed with reduction buffer (final concentration, 4% SDS, 0.1 M DTT, 0.1 M Tris-Cl, pH 7.4). After heating at 95℃ for 15 min, the reduced proteins were digested by filter-aided sample preparation (FASP), as previously described with some modification^1-5^. Briefly, the proteins were loaded onto a 30K spin filter (Millipore, Billerica, MA) and the buffer was exchanged three times with UA solution (8 M urea in 0.1 M Tris-Cl, pH 8.5) by centrifugation. The reduced cysteines were alkylated with 0.05 M iodoacetamide (IAA) in UA solution for 30 min at room temperature in the dark. The UA buffer was then exchanged with 40 mM ammonium bicarbonate (ABC) and the proteins were digested with trypsin (enzyme-to-substrate ratio, 1:100) at 37°C for 18 hrs. The digested peptides were acidified by trifluoroacetic acid and cleaned using in-house-generated C18 stagetips, as described^1,2,6^. Finally, we separated the desalted peptides into six fractions using high-pH reverse-phase fractionation, as described before^2^.

**Mass spectrometry**

We separated the peptides on a Nanoflow Easy-nLC 1000 (Thermo Fisher Scientific, Waltham, MA) using a two-column system comprising a trap column (Thermo Fisher Scientific, 75 μm I.D. x 2 cm long, 100 Å, 3 μm Acclaim PepMap100 C18 beads) and an analytical column (Thermo Fisher Scientific, 100 Å, 1.8-mm particle, 50 mm x 15 cm) as previously described with some modifications ^5^. A gradient from 5% to 30% acetonitrile was passed into the columns over 3 hrs at 300 nl/min. The eluted peptides were ionized using a spray voltage of 2.00 kV at the tip of column. The ionized peptides were analyzed using a quadrupole Orbitrap mass spectrometer (Q-Exactive; Thermo Fisher Scientific) in the data-dependent acquisition mode. The MS1 spectra were measured at a resolution of 70,000 and an automated gain control (AGC) target of 3.0E6. We set the instrumental parameters as follows; selection of the 20 most abundant ions with an isolation window of 2 m/z, fragmentation by higher-energy collisional dissociation (HCD) with a normalized collision energy of 27, and a resolution of 17,500 at 200 m/z. The dynamic exclusion of detected peptides was fixed to 30 s to restrict repeated analyzing. The maximum ion injection time for the analysis scan and MS2 scan were 20 ms and 60 ms, respectively. Each sample was analyzed in duplicate for technical replicates.

**Data analysis**

The raw MS files were processed using the peptide identification algorithm of the Maxquant software (ver. 1.5.3.12). The Andromeda search engine^7^ was used to search spectra against the UniprotKB FASTA database (82,074 entries included two human genes, *APP* and *PSEN1*, version from October 2014). MS/MS searches were performed with the following parameters: Peptides had to have a minimum length of six amino acids to be considered for identification. Carbamidomethylation was set as a fixed modification, while oxidation of methionine and protein N-terminal acetylation were set as variable modifications. The enzyme specificity was set to trypsin and allowed up to two miscleavages. The main and first search tolerances were set at 6 ppm and 20 ppm, respectively. A false discovery rate (FDR) of 1% was applied for all proteins and peptides. The retention times of all analyzed samples were linearized in Maxquant using the “Match between runs” function, which allowed transfer of identified peptides across the raw MS files to perform stable quantification even when a portion of MS/MS spectra were absent. The retention time window of 2 min. All proteins were filtered for common contaminants, such as keratins.

**Label-free quantification**

The data obtained from out Maxquant analyses were imported into the Perseus program^8^ for post-search analysis. The iBAQ intensity data were normalized using Tukey’s bi-weight method and filtered rows with at least three valid values per group. Proteins that showed significantly differential expression were filtered by ANOVA with permutations based on a FDR<0.01. A hierarchical clustering of the DEPs was performed based on Euclidean distance with k-means algorithm using the Perseus program.

**Bioinformatics** Gene Ontology (GO) analysis was performed using the DAVID bioinformatics tool ^9^. The GO classifications were evaluated by Fisher’s exact test to obtain p-values, which were filtered at a cut-off value of 0.05. Canonical pathways, downstream biological functions, and TreeMaps for disease and function were analyzed/generated using Ingenuity Pathway Analysis software (IPA; QIAGEN)^10^. The analytical algorithms embedded in the IPA software use lists of the differentially expressed proteins (DEPs) to predict biological processes and pathways. The IPA software also assigns activation states to putative regulators or pathways based on the quantitative values of the proteins. The p-value was obtained using Fisher’s exact test, and the magnitude of activation was given as a Z-score; the cut-off values used to determine enrichment (p-value) and predict activation (Z-score) were 0.05 and 1, respectively. For network analysis, the protein-protein interactions (PPIs) of the DEPs were interrogated from the STRING database^11^. The PPIs were visualized as a network model, which was generated using the Cytoscape software (version 3.1).

**Participants**

A total of 195 participants (67 cognitively normal, 60 mild cognitive impairment, 68 AD dementia) were included in this study. Details of criteria for clinical diagnosis are described previously^12^. They underwent clinical assessment by trained psychiatrists according to the Korean brain aging study for early diagnosis and prediction of Alzheimer’s disease (KBASE) protocol^13^. The study protocol was approved by the Institutional Review Board (IRB) of the Seoul National University Hospital and SMG-SNU Boramae Medical Center, South Korea. All participants or their legal representatives provided written informed consent.

**Neuroimaging data**

Participants underwent PET‑MR scanning sessions to obtain simultaneous three‑dimensional (3D) [^11^C] Pittsburgh compound B (PiB)‑Positron emission tomography (PET) and 3D T1‑weighted magnetic resonance imaging (MRI) using the 3.0T PET‑MR scanner (Siemens, Washington, DC, USA) according to the manufacturer’s approved guidelines.

**MRI acquisition and processing**

T1‑weighted images were acquired as follows: repetition time = 1670 ms, echo time = 1.89 ms, field of view 250 mm, and 256 X 256 matrix with 1.0‑mm slice thickness. All MR images were segmented using FreeSurfer version 6.0 (<http://surfer.nmr.mgh.harvard.edu/>)^14^. Adjusted hippocampal volume (Hva) was generated by using the residuals from linear regression of hippocampal volume against total intracranial volume of young normal controls as the reference group (age range=20‑55)^15^ and it is interpreted as the deviation in mm^3^. Details on image acquisition and processing were described previously^12,16^.

**PiB‑PET acquisition and processing**

After intravenous administration of 555 MBq of [^11^C]PiB (range, 450–610 MBq), a 30‑min emission scan was obtained 40 min after injection. The PiB‑PET data were processed for the several corrections such as decay correction, uniformity, and were re-constructed into a 256×256 image matrix using iterative methods. The automatic anatomic labeling algorithm and a region-combining method were applied to determine regions of interests (ROIs), to characterize the PiB retention levels in the frontal, lateral parietal, posterior cingulate-precuneus, and lateral temporal regions. The standardized uptake value ratio (SUVR) values for each ROI were calculated by dividing the mean value for all voxels within each ROI by the mean cerebellar uptake value in the same image. A global cortical ROI consisting of four ROIs was also defined and a global amyloid retention value was generated by dividing the mean value for all voxels of the global cortical ROI by the mean cerebellar uptake value in the same image. Each participant was categorized as PiB-positive (or Aβ-positive) if the SUVR value was > 1.4 in at least one of the four ROIs or as PiB-negative (or Aβ-negative) if the SUVR values of all four ROIs was ≤ 1.4^17^. Details on full acquisition and processing were described previously^12,16^.

**Blood sampling**

Overnight fasting blood samples (70 mL) were collected in the morning (9:00 AM) in SST serum separation tubes (BD, Franklin Lakes, NJ, USA), and centrifuged (3000g, 10 min, room temperature) to obtain serum supernatants. They were further centrifuged under the same conditions to obtain samples with high quality. The collected pure serum supernatants were aliquoted and stored at -80°C. For mouse serum, over 100 μl blood is collected from retro-orbital sinus and allowed to clot for 30 min at room temperature. To remove the clot, whole blood is centrifuged at 14,000g for 20min at 4℃^18^. The resulting supernatants were diluted with PBS (1:20 ratio).

**Immunohistochemistry (IHC)**

Mice were anesthetized and then perfused with phosphate-buffered saline (PBS) and 4% paraformaldehyde (PFA) solution to remove blood and fix brains. The brains were fixed in 4% PFA solution for 20 hrs at 4℃, followed by being incubated with 30% sucrose (wt/vol) solution diluted in PBS for 72 hrs at 4℃. The frozen tissue sections were washed with PBS and incubated in blocking solution containing 0.3 % Triton X-100, 5% horse serum, and 0.05% BSA solution for 1 hr. In the case of amyloid plaques staining, brain sections were incubated in 70% formic acid solution diluted in PBS for 20 min for antigen retrieval. Brain sections were incubated with a primary antibody overnight, such as biotin-4G8 antibody (1:700, Biolegend) for amyloid plaque staining and Protein S (Pros1) antibody (1:200, Santa Cruz), and then incubated for 1 hr with the appropriate secondary antibody, such as streptavidin-488-conjugated antibody (1:500, Life Technologies) and anti-Goat-Alexa594 antibody (1:500, Life Technologies). The results were visualized under a microscope (Zeiss), and image analysis was performed using the Image J software.

**Trichloroacetic acid (TCA) precipitation**

To investigate the proteins secreted to conditioned media, TCA precipitation was performed as previously described ^19^. Briefly, different cells lines were treated with Aβ in serum-free Opti-MEM. A sample of each conditioned medium was centrifuged at 13,000 rpm for 5 min, and the supernatant was mixed with TCA solution (Sigma-Aldrich, T6399), incubated overnight in a 4℃ rotator, and centrifuged at 13,000 rpm for 10 min at 4℃. The protein pellet was washed with 100% ice-cold acetone solution, and centrifuged at 13,000 rpm for 5 min at 4℃. The pellet was air-dried at 85℃ for 3 min and boiled with sample buffer for 5 min, and the proteins were processed for Western blot analysis.

**Western blot analysis**

At 5 and 10 months of age, mice were perfused with PBS and whole hippocampi were dissected. Each full sample was homogenized with RIPA buffer containing protease inhibitors, phosphatase inhibitors, and PMSF. The homogenized tissue sample was centrifuged for 15 min at 13,000 rpm. The supernatant was quantified using the BCA assay. Human blood serum was diluted in PBS (1:20), and 4 μl of the diluted serum samples was used without the BCA assay. Serum albumin protein was used as a loading control for human blood sera. Equal amounts of hippocampal samples were loaded to a 4-12% Bis-Tris polyacrylamide precast gel (NuPAGE system; Invitrogen, Carlsbad, CA, USA) and resolved by electrophoresis. The resolved proteins were transferred to a PVDF (polyvinylidene difluoride) membrane. The membrane was incubated with 5% skim milk solution for 1 hr and then incubated overnight with the relevant primary antibodies. The following primary antibodies were used: anti-PROS1 (1:1000, Proteintech), anti-S100b (1:1000, Cell Signaling Technology), anti-STAT3 (1:500, Santa Cruz), anti-β-actin (1:2000, Sigma Aldrich), anti-GFAP (1:1000, Invitrogen), anti-CDK5 (1:1000, Cell Signaling Technology), and anti-GRM2 (1:1000, Millipore). Membranes were incubated with secondary antibodies, and then developed with an ECL solution (West Save Gold; Ab Frontier Co., Seoul, Korea).

**Real-time PCR analysis**

To measure the mRNA levels of PROS1 in different cell types, real-time PCR analysis was performed. Total RNA was isolated with an RNeasy Mini kit (QIAGEN) and converted into cDNA using a Maxime RT preMix kit (iNtRON Biotech Co., Seoul, Korea). Quantitative real-time PCR was carried out with KAPA SYBR FAST ABI Prism qPCR kit (KAPA biosystems). The utilized primers were as follows: PROS1, 5'-GCA CAG TGC CCT TTG CCT-3' and 5'-CAA ATA CCA CAA TAT CCT GAG ACG TT-3'; and 18s rRNA (used as a normalization control), 5'-GTA ACC CGT TGA ACC CCA TT-3' and 5'-CCA TCC AAT CGG TAG TAG CG-3'.

**Microglial phagocytosis assay**

Microglial phagocytosis assay was performed as previously described^20^. GFP overexpressing HT22 hippocampal neuronal cells were treated with 2 μΜ staurosporine (Sigma Aldrich) for 4 hrs to induce apoptosis. After wash with PBS, equal volumes of apoptotic cell suspension (100 μl) were added to primary microglia with Aβ (2 μΜ) or recombinant mouse PROS1 (30nM, R&D systems, USA) for 24 hrs. After incubation with the apoptotic cell suspension, primary microglia were fixed with 4% paraformaldehyde solution for 15 min and then incubated with anti-GFP (1:500, Abcam) and Iba-1 antibodies (1:1000, Wako) overnight at 4℃. Microglia were washed with PBS followed by incubation with secondary antibodies (1:500, Alexa488 and 594 antibodies, Invitrogen) for 1 hr at room temperature. Microglia were visualized with Zeiss LSM700 confocal microscope.

**Intrahippocampal injection of clodronate liposome**

To deplete local microglia in the hippocampus, control liposomes or clodronate liposomes (Anionic, 7 mg/ml, Formumax F70101C-A) were administered into 15-month old wild-type or 5XFAD mice. After mice were anesthetized, 2 μl of control liposomes or clodronate liposomes were bilaterally injected into the hippocampus (AP, -2.0 mm; ML, ±1.3 mm; DV, 2.0 mm from bregma). 4 and 6 days following administration of control liposomes or clodronate liposomes, blood serum was collected to examine serum PROS1 levels by western blot analysis.

**References**

1. Han, D.*, et al.* In-depth proteomic analysis of mouse microglia using a combination of FASP and StageTip-based, high pH, reversed-phase fractionation. *Proteomics* **13**, 2984-2988 (2013).

2. Han, D., Jin, J., Woo, J., Min, H. & Kim, Y. Proteomic analysis of mouse astrocytes and their secretome by a combination of FASP and StageTip-based, high pH, reversed-phase fractionation. *Proteomics* **14**, 1604-1609 (2014).

3. Woo, J., Han, D., Park, J., Kim, S.J. & Kim, Y. In-depth characterization of the secretome of mouse CNS cell lines by LC-MS/MS without prefractionation. *Proteomics* **15**, 3617-3622 (2015).

4. Woo, J.*, et al.* Quantitative Proteomics Reveals Temporal Proteomic Changes in Signaling Pathways during BV2 Mouse Microglial Cell Activation. *J Proteome Res* **16**, 3419-3432 (2017).

5. Kim, D.K.*, et al.* Molecular and functional signatures in a novel Alzheimer's disease mouse model assessed by quantitative proteomics. *Mol Neurodegener* **13**, 2 (2018).

6. Park, J.*, et al.* Proteome characterization of human pancreatic cyst fluid from intraductal papillary mucinous neoplasm by liquid chromatography/tandem mass spectrometry. *Rapid Commun Mass Spectrom* **31**, 1761-1772 (2017).

7. Tyanova, S., Temu, T. & Cox, J. The MaxQuant computational platform for mass spectrometry-based shotgun proteomics. *Nat Protoc* **11**, 2301-2319 (2016).

8. Tyanova, S.*, et al.* The Perseus computational platform for comprehensive analysis of (prote)omics data. *Nat Methods* **13**, 731-740 (2016).

9. Huang da, W., Sherman, B.T. & Lempicki, R.A. Systematic and integrative analysis of large gene lists using DAVID bioinformatics resources. *Nat Protoc* **4**, 44-57 (2009).

10. Kramer, A., Green, J., Pollard, J., Jr. & Tugendreich, S. Causal analysis approaches in Ingenuity Pathway Analysis. *Bioinformatics* **30**, 523-530 (2014).

11. Szklarczyk, D.*, et al.* STRING v10: protein-protein interaction networks, integrated over the tree of life. *Nucleic Acids Res* **43**, D447-452 (2015).

12. Byun, M.S.*, et al.* Korean Brain Aging Study for the Early Diagnosis and Prediction of Alzheimer's Disease: Methodology and Baseline Sample Characteristics. *Psychiatry Investig* **14**, 851-863 (2017).

13. Lee, J.H.*, et al.* Development of the Korean version of the Consortium to Establish a Registry for Alzheimer's Disease Assessment Packet (CERAD-K): clinical and neuropsychological assessment batteries. *J Gerontol B Psychol Sci Soc Sci* **57**, P47-53 (2002).

14. Desikan, R.S.*, et al.* An automated labeling system for subdividing the human cerebral cortex on MRI scans into gyral based regions of interest. *Neuroimage* **31**, 968-980 (2006).

15. Jack, C.R., Jr.*, et al.* Age-specific population frequencies of cerebral beta-amyloidosis and neurodegeneration among people with normal cognitive function aged 50-89 years: a cross-sectional study. *Lancet Neurol* **13**, 997-1005 (2014).

16. Park, J.C.*, et al.* Plasma tau/amyloid-beta1-42 ratio predicts brain tau deposition and neurodegeneration in Alzheimer's disease. *Brain* **142**, 771-786 (2019).

17. Villeneuve, S.*, et al.* Existing Pittsburgh Compound-B positron emission tomography thresholds are too high: statistical and pathological evaluation. *Brain* **138**, 2020-2033 (2015).

18. Greenfield, E.A. Sampling and Preparation of Mouse and Rat Serum. *Cold Spring Harb Protoc* **2017**, pdb prot100271 (2017).

19. Son, S.M.*, et al.* Altered APP processing in insulin-resistant conditions is mediated by autophagosome accumulation via the inhibition of mammalian target of rapamycin pathway. *Diabetes* **61**, 3126-3138 (2012).

20. Chauss, D., Brennan, L.A., Bakina, O. & Kantorow, M. Integrin alphaVbeta5-mediated Removal of Apoptotic Cell Debris by the Eye Lens and Its Inhibition by UV Light Exposure. *J Biol Chem* **290**, 30253-30266 (2015).
