# Supplementary material for: Deep proteome profiling of the hippocampus in the 5XFAD mouse model reveals biological process alterations and a novel biomarker of Alzheimer’s disease
Source: Exp Mol Med. 2019 Nov 15;51(11):136. doi: 10.1038/s12276-019-0326-z (PMC6856180; doi:10.1038/s12276-019-0326-z)
Supplement: Supplementary file 2 — Supplementary figure [file 12276_2019_326_MOESM2_ESM.pptx]

## Slide 1
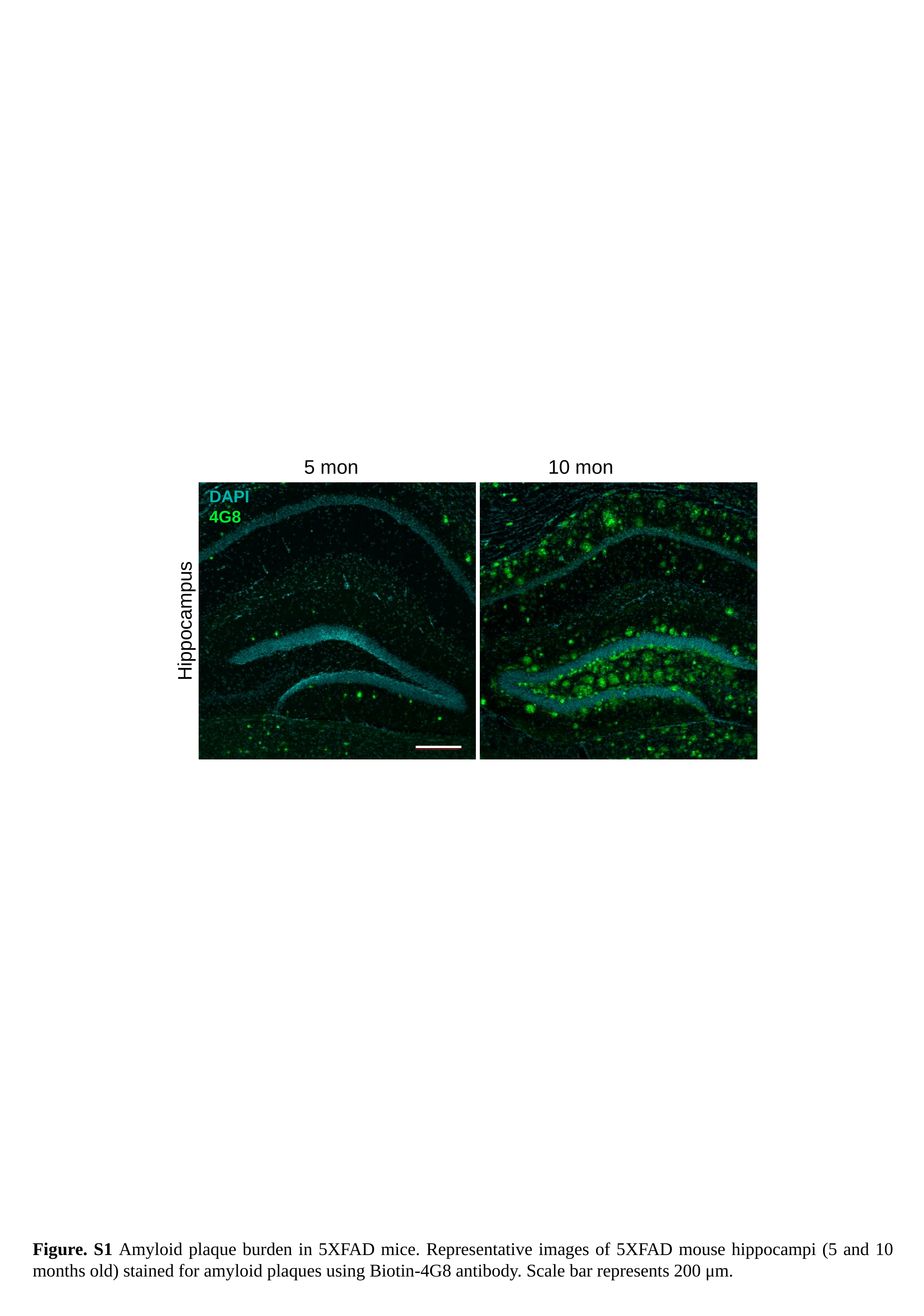

5 mon	 10 mon
DAPI
4G8
Hippocampus
Figure. S1 Amyloid plaque burden in 5XFAD mice. Representative images of 5XFAD mouse hippocampi (5 and 10 months old) stained for amyloid plaques using Biotin-4G8 antibody. Scale bar represents 200 μm.

## Slide 2
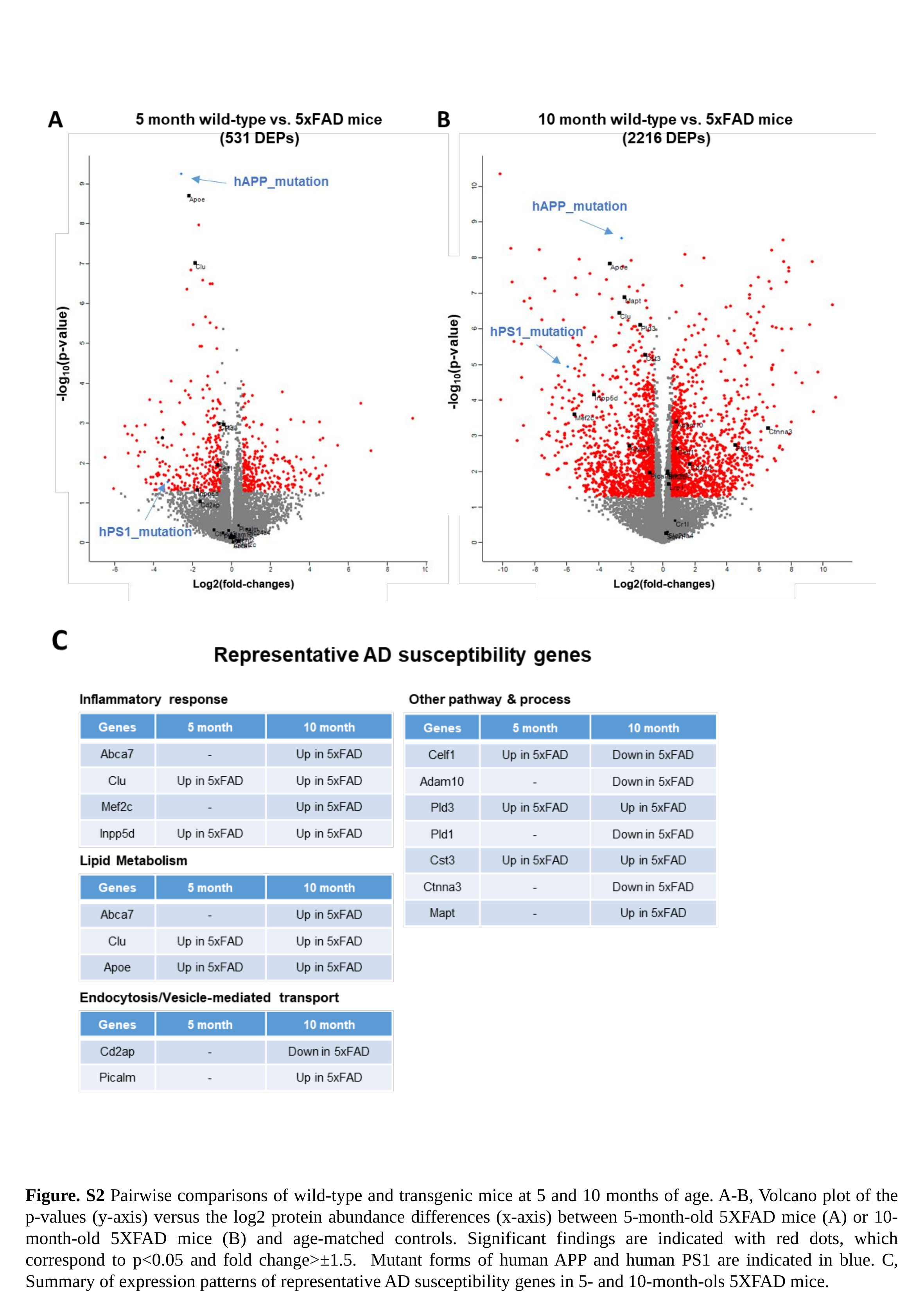

Figure. S2 Pairwise comparisons of wild-type and transgenic mice at 5 and 10 months of age. A-B, Volcano plot of the p-values (y-axis) versus the log2 protein abundance differences (x-axis) between 5-month-old 5XFAD mice (A) or 10-month-old 5XFAD mice (B) and age-matched controls. Significant findings are indicated with red dots, which correspond to p<0.05 and fold change>±1.5. Mutant forms of human APP and human PS1 are indicated in blue. C, Summary of expression patterns of representative AD susceptibility genes in 5- and 10-month-ols 5XFAD mice.

## Slide 3
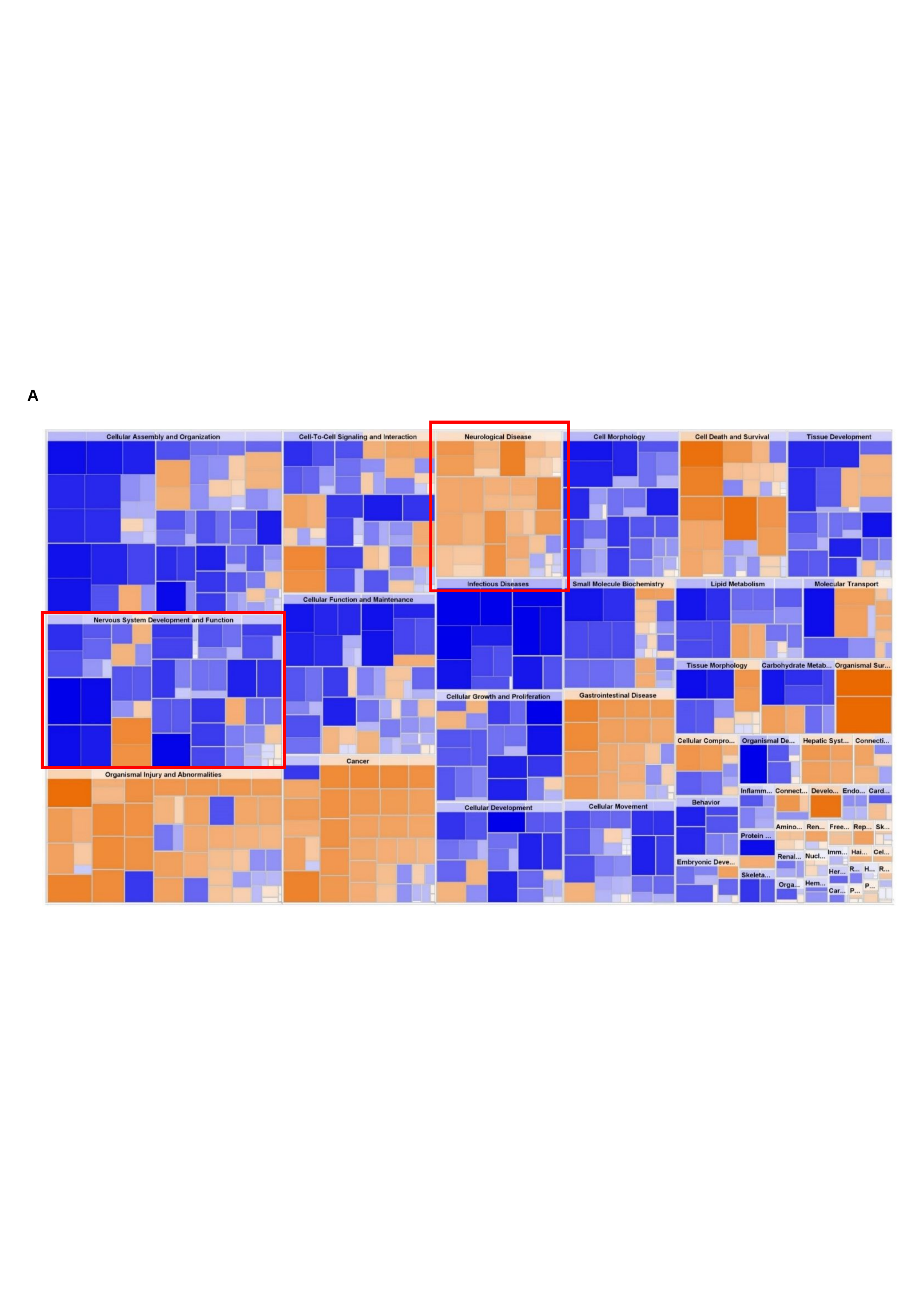

A

## Slide 4
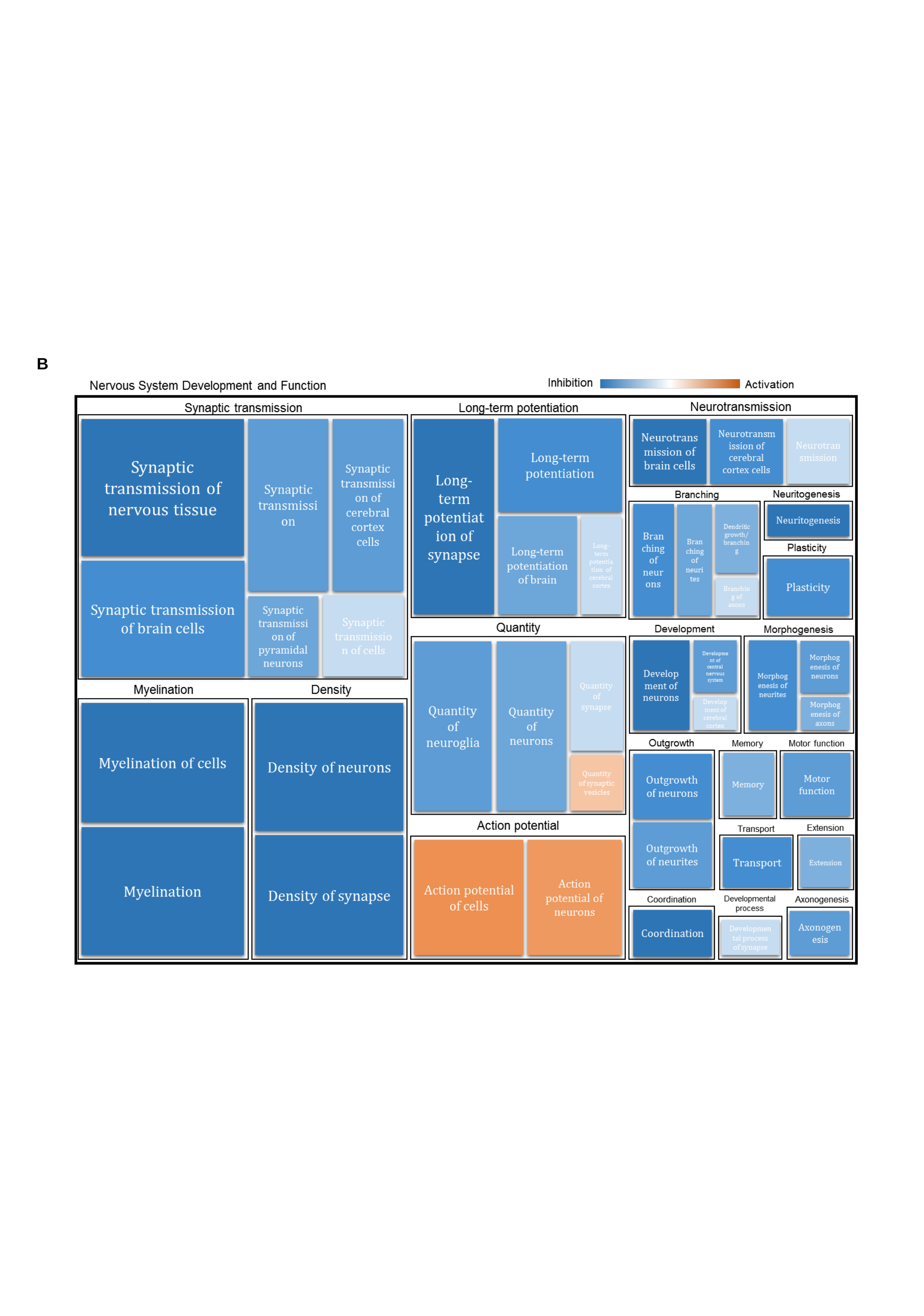

B

## Slide 5
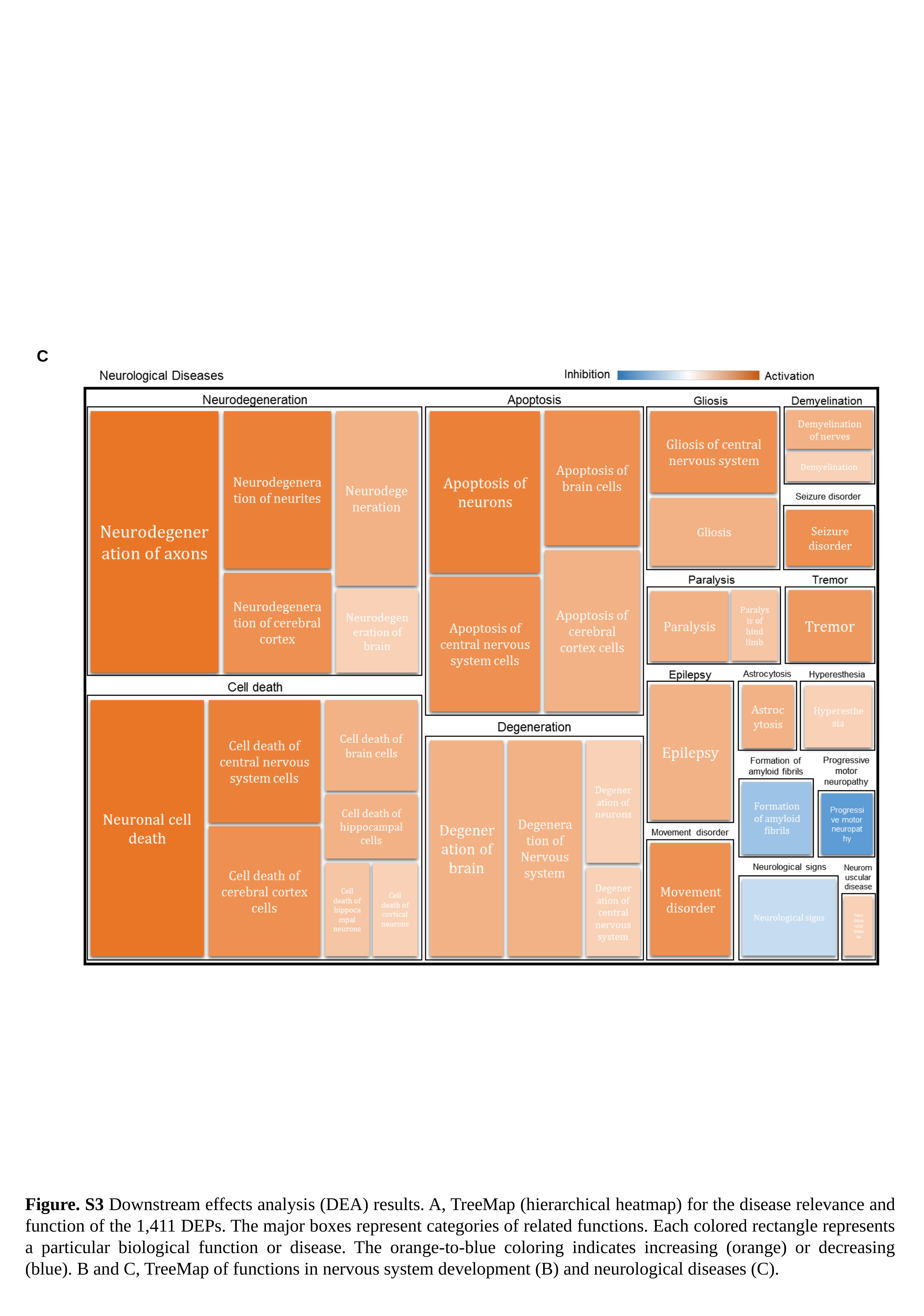

C
Figure. S3 Downstream effects analysis (DEA) results. A, TreeMap (hierarchical heatmap) for the disease relevance and function of the 1,411 DEPs. The major boxes represent categories of related functions. Each colored rectangle represents a particular biological function or disease. The orange-to-blue coloring indicates increasing (orange) or decreasing (blue). B and C, TreeMap of functions in nervous system development (B) and neurological diseases (C).

## Slide 6
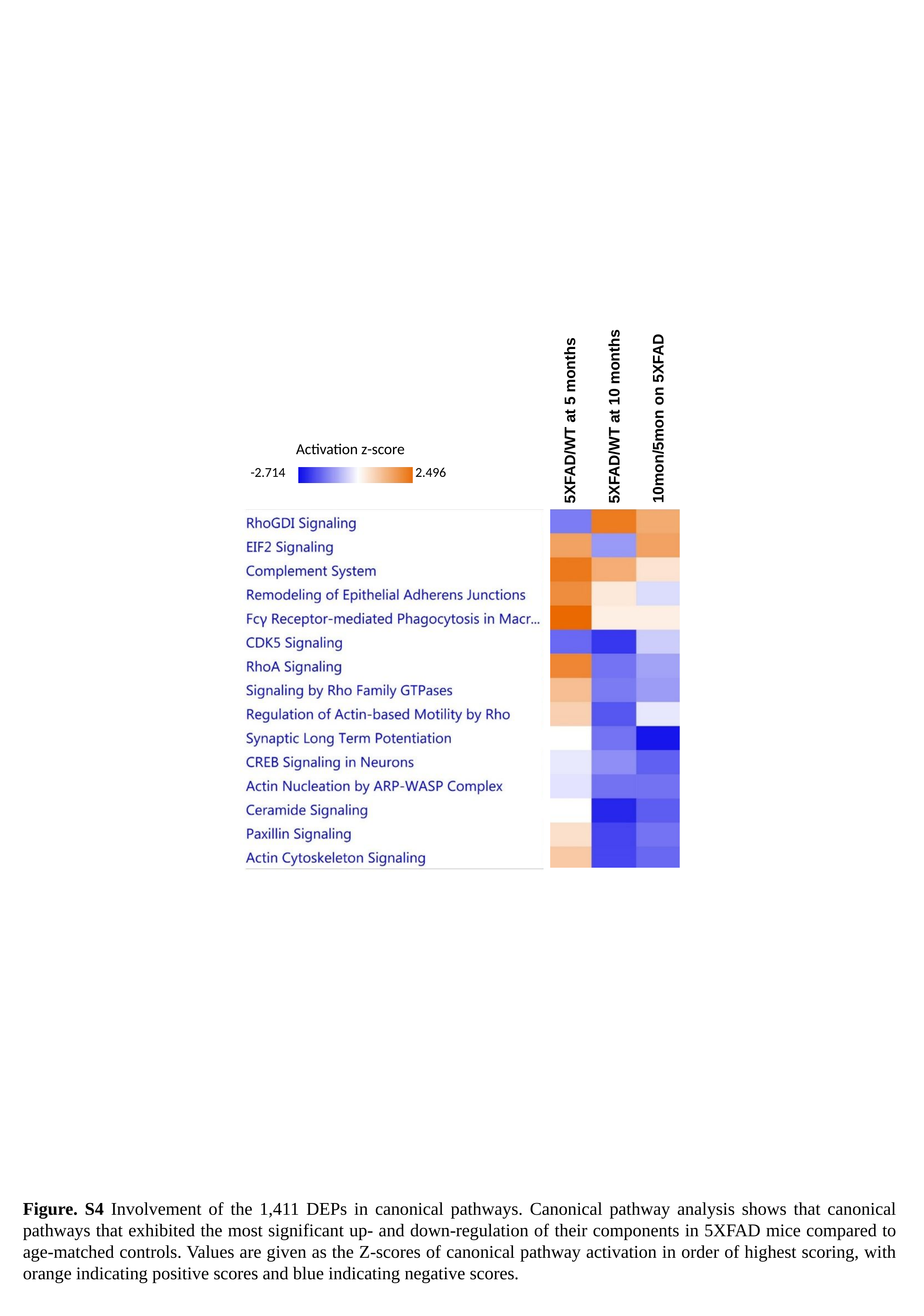

5XFAD/WT at 10 months
10mon/5mon on 5XFAD
5XFAD/WT at 5 months
Activation z-score
-2.714	 2.496
Figure. S4 Involvement of the 1,411 DEPs in canonical pathways. Canonical pathway analysis shows that canonical pathways that exhibited the most significant up- and down-regulation of their components in 5XFAD mice compared to age-matched controls. Values are given as the Z-scores of canonical pathway activation in order of highest scoring, with orange indicating positive scores and blue indicating negative scores.

## Slide 7
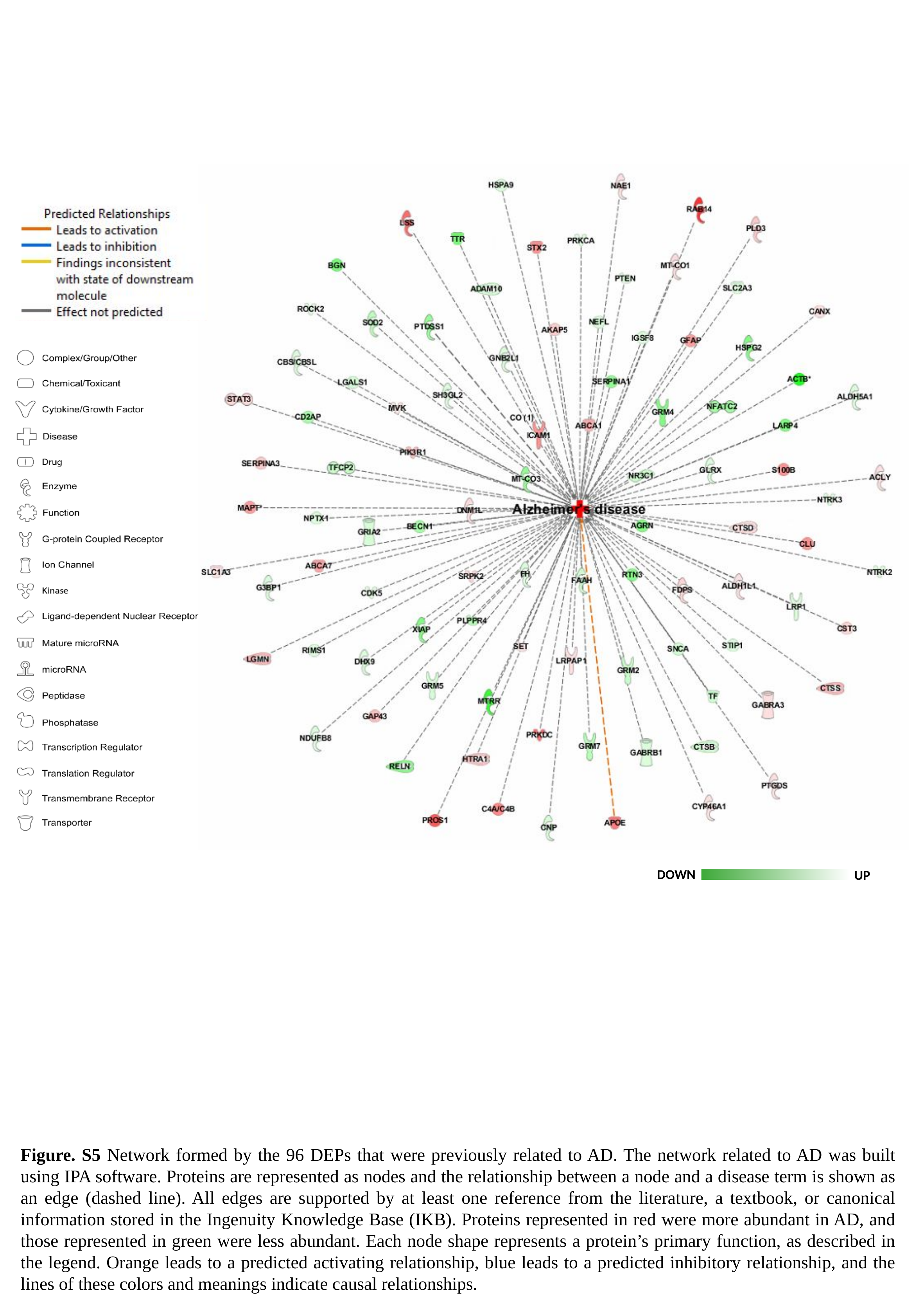

DOWN
UP
Figure. S5 Network formed by the 96 DEPs that were previously related to AD. The network related to AD was built using IPA software. Proteins are represented as nodes and the relationship between a node and a disease term is shown as an edge (dashed line). All edges are supported by at least one reference from the literature, a textbook, or canonical information stored in the Ingenuity Knowledge Base (IKB). Proteins represented in red were more abundant in AD, and those represented in green were less abundant. Each node shape represents a protein’s primary function, as described in the legend. Orange leads to a predicted activating relationship, blue leads to a predicted inhibitory relationship, and the lines of these colors and meanings indicate causal relationships.

## Slide 8
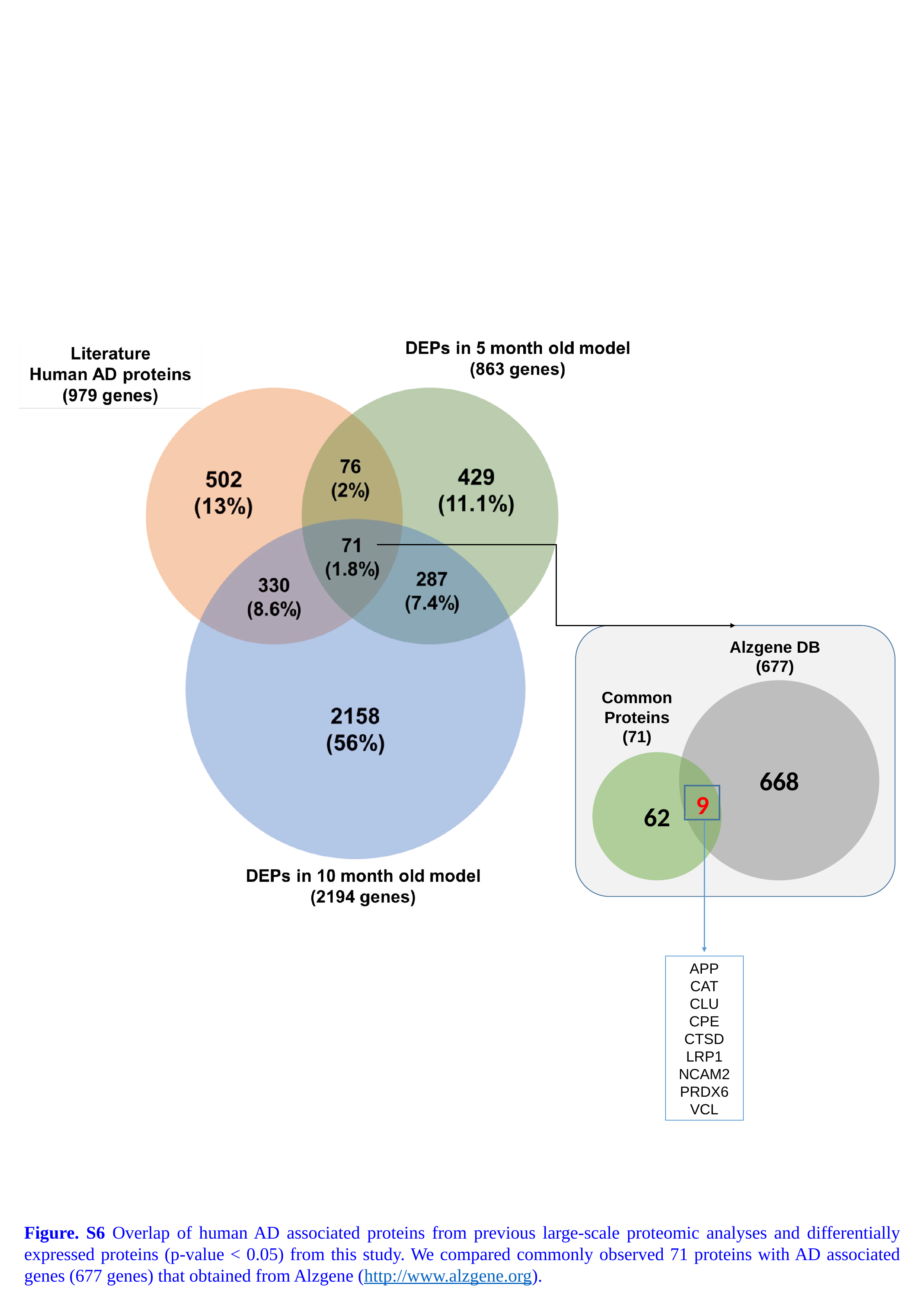

Alzgene DB
(677)
Common
Proteins
(71)
668
9
62
APP
CAT
CLU
CPE
CTSD
LRP1
NCAM2
PRDX6
VCL
Figure. S6 Overlap of human AD associated proteins from previous large-scale proteomic analyses and differentially expressed proteins (p-value < 0.05) from this study. We compared commonly observed 71 proteins with AD associated genes (677 genes) that obtained from Alzgene (http://www.alzgene.org).

## Slide 9
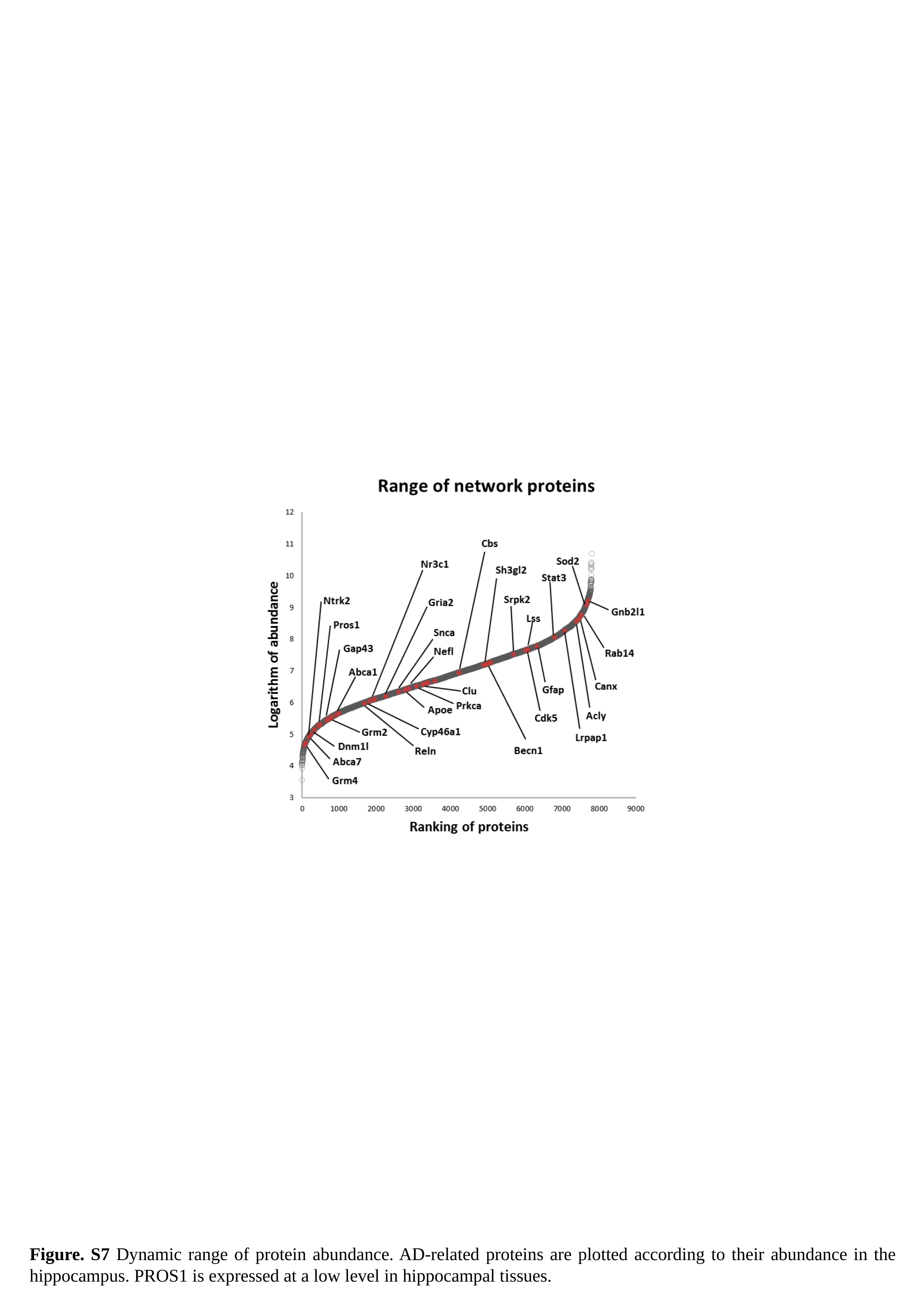

Figure. S7 Dynamic range of protein abundance. AD-related proteins are plotted according to their abundance in the hippocampus. PROS1 is expressed at a low level in hippocampal tissues.

## Slide 10
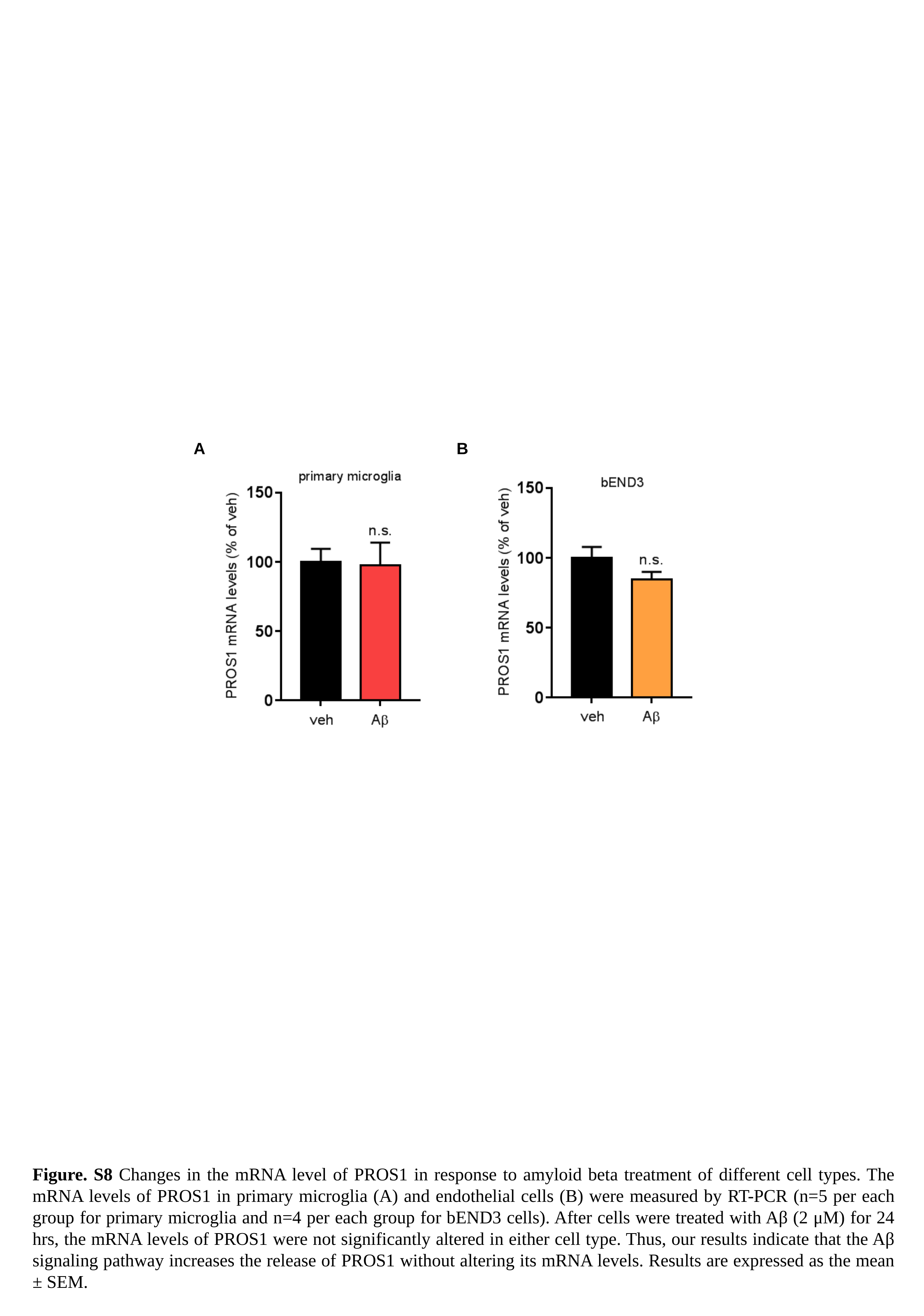

A
B
Figure. S8 Changes in the mRNA level of PROS1 in response to amyloid beta treatment of different cell types. The mRNA levels of PROS1 in primary microglia (A) and endothelial cells (B) were measured by RT-PCR (n=5 per each group for primary microglia and n=4 per each group for bEND3 cells). After cells were treated with Aβ (2 μM) for 24 hrs, the mRNA levels of PROS1 were not significantly altered in either cell type. Thus, our results indicate that the Aβ signaling pathway increases the release of PROS1 without altering its mRNA levels. Results are expressed as the mean ± SEM.

## Slide 11
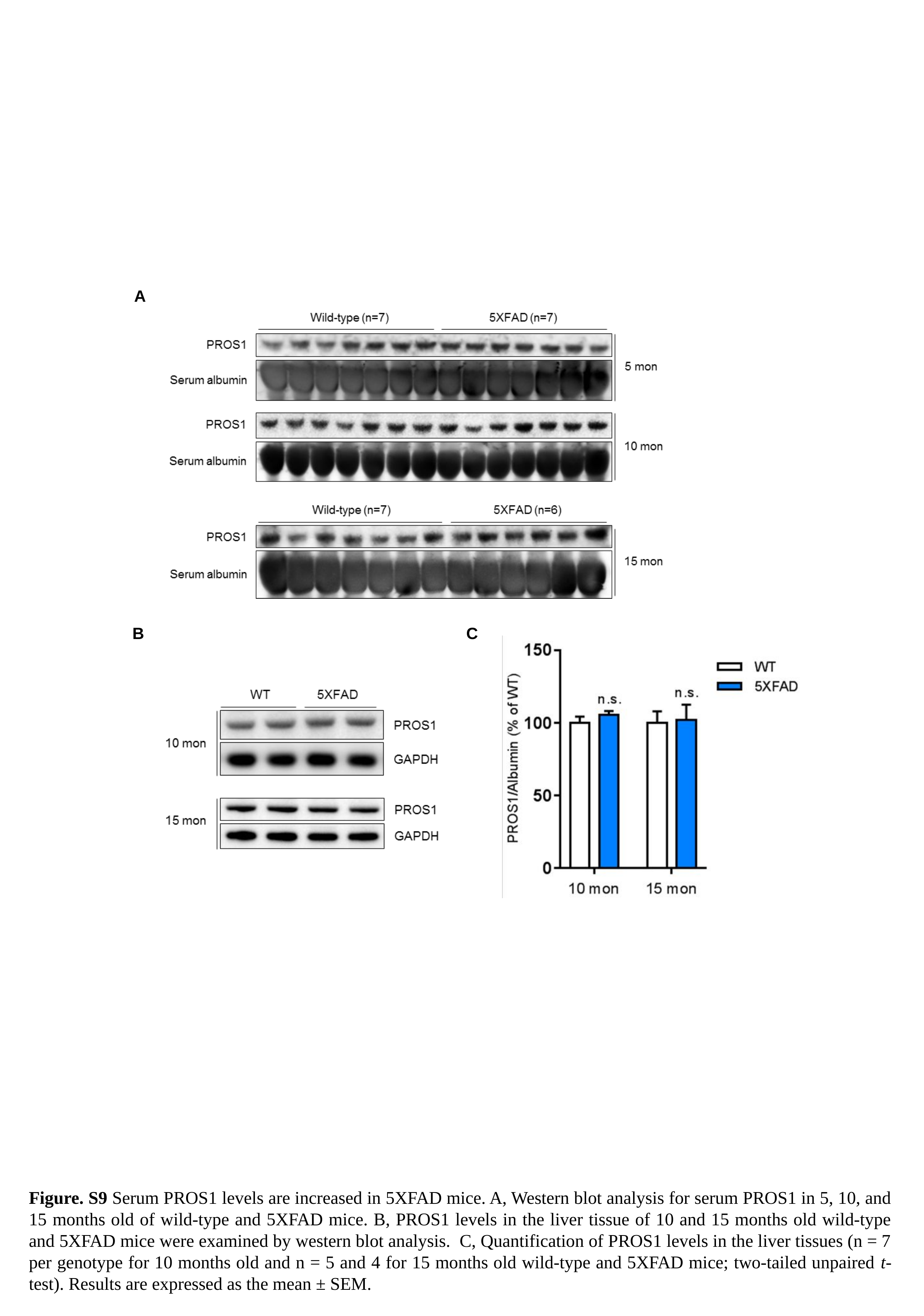

A
B
C
Figure. S9 Serum PROS1 levels are increased in 5XFAD mice. A, Western blot analysis for serum PROS1 in 5, 10, and 15 months old of wild-type and 5XFAD mice. B, PROS1 levels in the liver tissue of 10 and 15 months old wild-type and 5XFAD mice were examined by western blot analysis. C, Quantification of PROS1 levels in the liver tissues (n = 7 per genotype for 10 months old and n = 5 and 4 for 15 months old wild-type and 5XFAD mice; two-tailed unpaired t-test). Results are expressed as the mean ± SEM.

## Slide 12
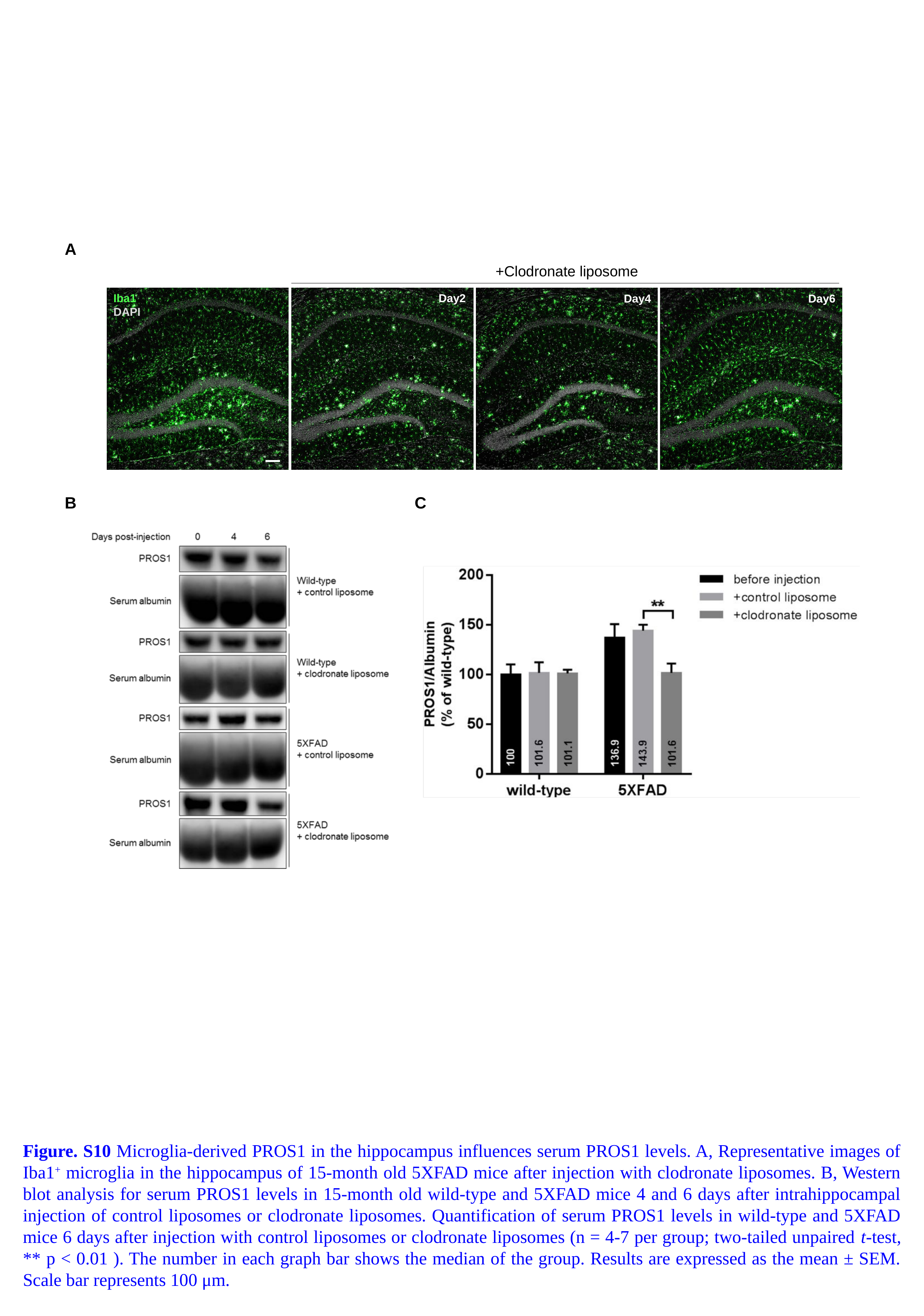

A
+Clodronate liposome
Iba1
DAPI
Day2
Day4
Day6
B
C
Figure. S10 Microglia-derived PROS1 in the hippocampus influences serum PROS1 levels. A, Representative images of Iba1+ microglia in the hippocampus of 15-month old 5XFAD mice after injection with clodronate liposomes. B, Western blot analysis for serum PROS1 levels in 15-month old wild-type and 5XFAD mice 4 and 6 days after intrahippocampal injection of control liposomes or clodronate liposomes. Quantification of serum PROS1 levels in wild-type and 5XFAD mice 6 days after injection with control liposomes or clodronate liposomes (n = 4-7 per group; two-tailed unpaired t-test, ** p < 0.01 ). The number in each graph bar shows the median of the group. Results are expressed as the mean ± SEM. Scale bar represents 100 μm.

## Slide 13
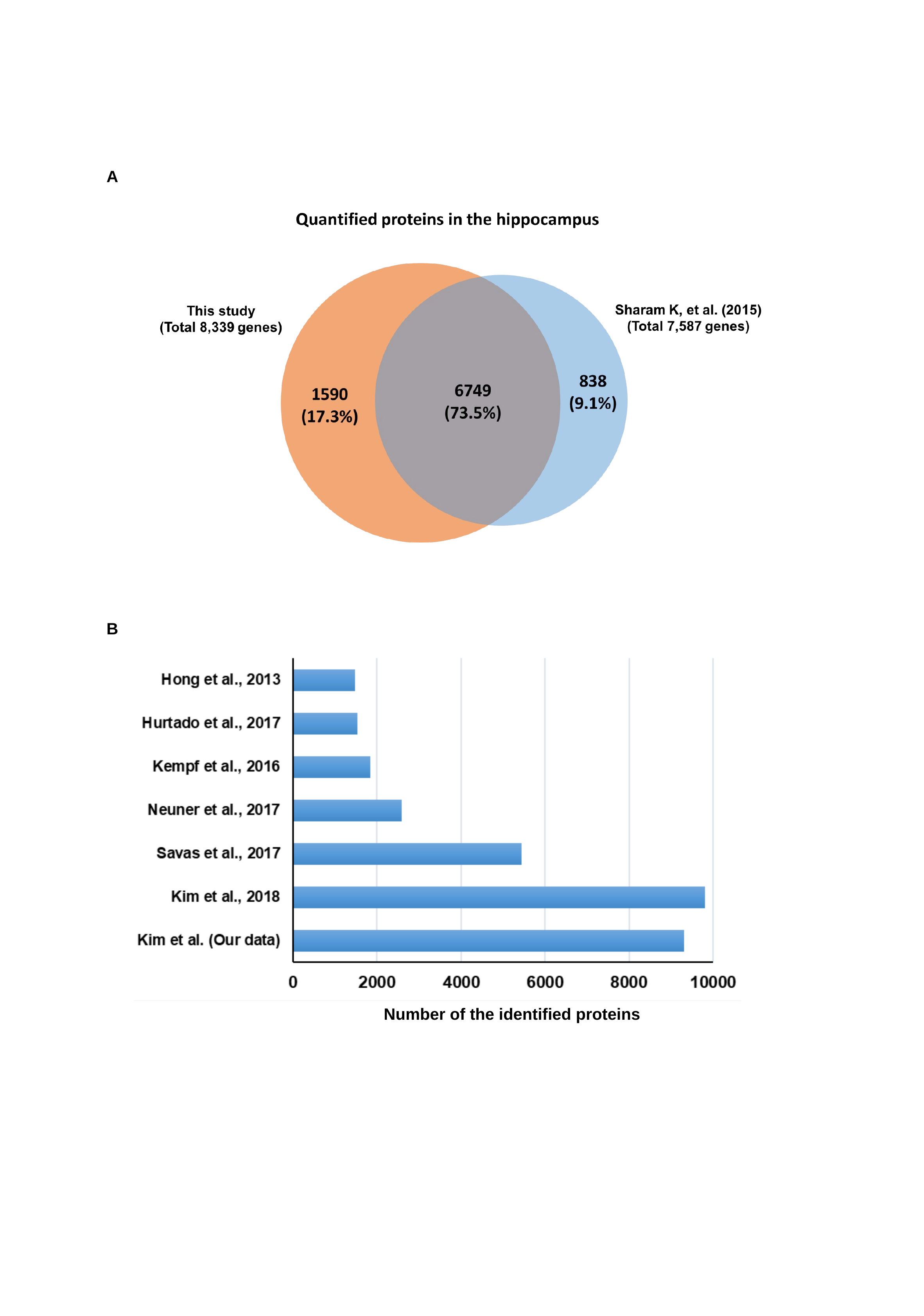

A
B
Number of the identified proteins

## Slide 14
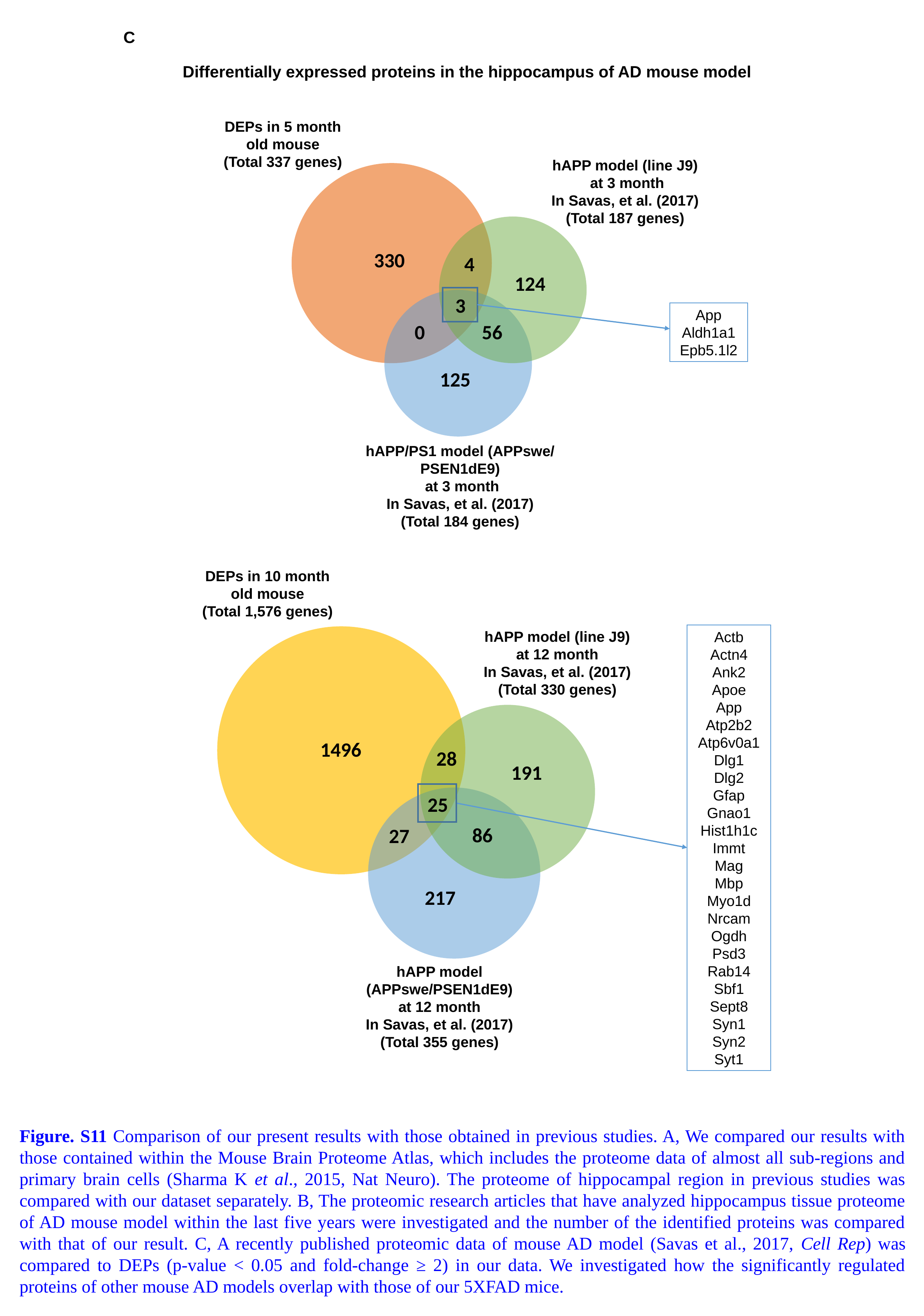

C
Differentially expressed proteins in the hippocampus of AD mouse model
DEPs in 5 month
old mouse
(Total 337 genes)
hAPP model (line J9)
 at 3 month
In Savas, et al. (2017)
(Total 187 genes)
330
4
124
3
App
Aldh1a1
Epb5.1l2
0
56
125
hAPP/PS1 model (APPswe/PSEN1dE9)
 at 3 month
In Savas, et al. (2017)
(Total 184 genes)
DEPs in 10 month
old mouse
(Total 1,576 genes)
hAPP model (line J9)
at 12 month
In Savas, et al. (2017)
(Total 330 genes)
Actb
Actn4
Ank2
Apoe
App
Atp2b2
Atp6v0a1
Dlg1
Dlg2
Gfap
Gnao1
Hist1h1c
Immt
Mag
Mbp
Myo1d
Nrcam
Ogdh
Psd3
Rab14
Sbf1
Sept8
Syn1
Syn2
Syt1
1496
28
191
25
86
27
217
hAPP model
(APPswe/PSEN1dE9)
at 12 month
In Savas, et al. (2017)
(Total 355 genes)
Figure. S11 Comparison of our present results with those obtained in previous studies. A, We compared our results with those contained within the Mouse Brain Proteome Atlas, which includes the proteome data of almost all sub-regions and primary brain cells (Sharma K et al., 2015, Nat Neuro). The proteome of hippocampal region in previous studies was compared with our dataset separately. B, The proteomic research articles that have analyzed hippocampus tissue proteome of AD mouse model within the last five years were investigated and the number of the identified proteins was compared with that of our result. C, A recently published proteomic data of mouse AD model (Savas et al., 2017, Cell Rep) was compared to DEPs (p-value < 0.05 and fold-change ≥ 2) in our data. We investigated how the significantly regulated proteins of other mouse AD models overlap with those of our 5XFAD mice.
